# From beer to breadboards: yeast as a force for biological innovation

**DOI:** 10.1186/s13059-023-03156-9

**Published:** 2024-01-04

**Authors:** Hamid Kian Gaikani, Monika Stolar, Divya Kriti, Corey Nislow, Guri Giaever

**Affiliations:** 1 Faculty of Pharmaceutical Sciences, University of British Columbia, Vancouver, BC Canada; 2 Department of Chemistry, University of British Columbia, Vancouver, BC Canada

## Abstract

The history of yeast *Saccharomyces cerevisiae*, aka brewer’s or baker’s yeast, is intertwined with our own. Initially domesticated 8,000 years ago to provide sustenance to our ancestors, for the past 150 years, yeast has served as a model research subject and a platform for technology. In this review, we highlight many ways in which yeast has served to catalyze the fields of functional genomics, genome editing, gene–environment interaction investigation, proteomics, and bioinformatics—emphasizing how yeast has served as a catalyst for innovation. Several possible futures for this model organism in synthetic biology, drug personalization, and multi-omics research are also presented.

**Supplementary Information:**

The online version contains supplementary material available at 10.1186/s13059-023-03156-9.

## A short history of yeast from the perspective of genomics

Tracing the journey of the budding yeast, *Saccharomyces cerevisiae* (*S. cerevisiae*) reveals a rich history of interactions with our ancestors and us. Domesticated for millennia for wine and breadmaking, yeast was introduced as an experimental organism in the 1930s by Herschel Roman and colleagues [1]. Genetic studies, pioneered by Øjvind Winge and Carl Lindegren in the late 1940s [2], helped set the stage for the broad adoption of this model system. In the ensuing century, *S. cerevisiae* became a workhorse in genetics, molecular biology, and biotechnology; more recently, it facilitated the establishment of genomics as a discipline. For example, *S. cerevisiae* has become a model for studying metabolism, morphology, cell division, secretion, and other fundamental cellular functions. Its experimental advantages are manifold—in particular, it is a unicellular organism that, unlike metazoans, can be cultured on defined media, allowing the researcher to control all environmental factors.

The introduction of genetically stable, homothallic strains (i.e., lacking a functional HO endonuclease and unable to switch mating type), genetically marked haploid and diploid cells, and the ability to control mating and meiosis opened up the field of classical yeast genetics. Both mitotic and meiotic approaches were developed to map yeast genes (reviewed in reference [3]). After the first genetic map was published in 1949 [4], molecular techniques and recombinant DNA—introduced in the 1950s and 1960s—were quickly adapted to yeast research. Later, a watershed experiment in 1977 was the demonstration of functional complementation of a yeast mutant with a leucine biosynthetic gene from *Escherichia coli* [5].

**Fig. 1:**
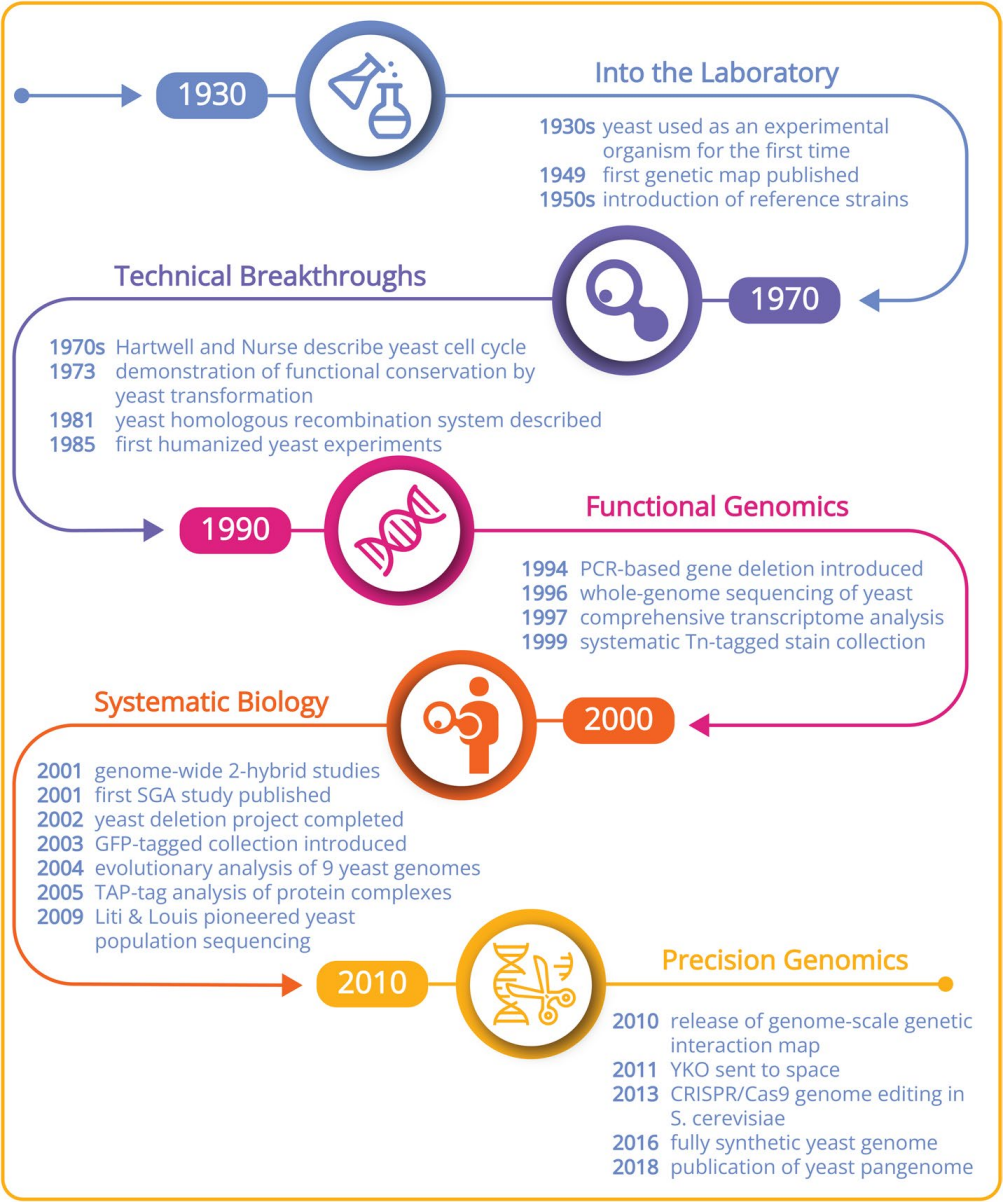
A timeline highlighting significant events or breakthroughs in yeast research (for details, see references [6–10]

Combining both genetic and molecular approaches to study yeast cells accelerated the development of reverse genetics (i.e., proceeding from gene to phenotype) and led to the characterization of hundreds of yeast genes. In parallel, yeast biochemists gathered a wealth of biochemical information on metabolic pathways, characterizing enzymes involved in metabolic processes as well as the underlying regulatory circuits. Cytological studies have contributed to our understanding of mitosis and meiosis, cytoskeletal structure and function, and organelle biology. Yeast studies have also contributed foundational insights on nucleic acid metabolism and genome structure, DNA repair, cell cycle regulation, gene expression, and the response to diverse stresses. Indeed, several Nobel Prizes have recognized these contributions to our understanding of the cell cycle, secretion, and autophagy (see Fig. 1 and reference [2] for details).

Because its core biological processes are functionally conserved, yeast research has direct, translational implications for human health [6]. Once sequence data became available on a large scale in the early 1990s, it became obvious that, despite being separated by nearly a billion years of evolutionary distance, most fundamental biological structures and functions are conserved between yeast and mammals. Indeed, many homologous genes can complement (i.e., functionally substitute) for each other (for instance, see reference [11]). By the 1990s, interest in the yeast genome was ascendant; as part of the Human Genome Project (HGP), the smaller genomes of yeast and worm served as pilot tests of HGP experimental and computational logistics (see reference [12] by Lander et al.). When *S. cerevisiae* became the first completely sequenced eukaryotic genome in 1996, the abundance of information collected in this project (performed by a network of yeast labs led by Andre Goffeau and colleagues) became a crucial reference against which human, animal, plant, and microbial genes were compared [13]. Despite the *S. cerevisiae* genome being one of the best-characterized and extensively studied model systems, it is somewhat surprising that several hundred open reading frames (ORFs) remain uncharacterized (Table 1). The yeast genome sequence revealed that between a third to a half of yeast genes are related to human genes by homology. The fact that these homologs have persisted (with modest alterations) suggests that they support important basic cellular functions (reviewed in reference [7]).

**Table 1:** Some key characteristics of *Saccharomyces cerevisiae* S288C (downloaded in August of 2023 from yeastmine.yeastgenome.org. The yeast genome has been revised multiple times since 1996, and a recent study using long-read, single molecule sequence provided a complete, telomere-to-telomere genome sequence [14]

Genomic feature	Count
Total ORFs	6605
Verified ORFs	5195
Uncharacterized ORFs	722
Dubious ORFs	688
Human genes complementing or complemented by yeast genes	599
Retrotransposon	50
Autonomously replicating sequences (ARSs)	352
Human genes with ortholog in yeast	2696 [15]

At the turn of the last century, comparative studies of a small number of closely related yeast genomes helped build the framework for comparative genomics [16]. The introduction of massively parallel sequencing brought hundreds (and eventually thousands) of diverse *S. cerevisiae* genomes for comparison [17–21]. In 2009, Gianni Liti and colleagues accelerated the nascent field of yeast population genetics by sequencing over 70 yeast isolates. They found that phenotypic variation was correlated with global genome-wide phylogenetic relationships. This study also revealed that human influences facilitated cross-breeding and the emergence of new variations [22]. Schacherer et al. conducted a nucleotide-level survey of genomic variation in 63 *S. cerevisiae* strains sampled from diverse ecological niches. They identified 1.89 million single-nucleotide polymorphisms and 3985 larger deletions. The study provided insights into the population structure of *S. cerevisiae*, supporting multiple domestication events, and also shed light on the origins of pathogenic strains [18]. In a study led by John H. McCusker, 93 genomes of *S. cerevisiae* strains from various geographic and environmental origins were sequenced and annotated as part of the “100-genomes” resource [23]. These studies set the stage for ever larger whole-genome surveys of *Saccharomyces*. For example, Peter et al. reported whole-genome sequencing and phenotyping of 1011 *S. cerevisiae* isolates, providing broad evolutionary insight into how genomic variants shape the species-wide phenotypic landscape [24], including evidence that *S. cerevisiae* spread worldwide from a single out-of-China event.

These comparative genomic projects, combined with the large-scale analysis of genetic regulatory elements and chromatin structure studies, provided the data that fueled a comprehensive annotation of the yeast genome, which continues to this day. Genome annotation describes the process of identifying the functional elements and characteristics of genes within a genome. In practice, gene and genome annotation involves several overlapping activities, including the following: (1) computational gene prediction to identify ORFs and noncoding regions, (2) functional annotation using both forward and reverse genetics, (3) identification of gene–gene interactions and gene–chemical interactions, (4) regulatory element identification, and (5) comparative genomics. These studies, which collectively assess over a thousand diverse species, provide a comprehensive view of genome evolution, including SNPs, structural changes, and large-scale differences in ploidy (i.e., changes in chromosome number). Most recently, long-read sequencing has been added to the toolkit of comparative genomics—enabling complete or nearly complete telomere-to-telomere genome assemblies [14].

The yeast sequencing project was contemporaneous with the establishment of the GO Consortium, which began as a joint project of the SGD [25], FlyBase [26], and the Mouse Genome Database [27]. The founders of the GO consortium envisioned gene annotation as a tool that would unify biology; their prediction that “there is likely to be a single limited universe of genes and proteins, many of which are conserved in most or all living cells,” has motivated a generation of computational biologists. For practical purposes, the consortium defined three categories of GO: *biological process*, i.e., the biological objective that the gene product executes; *molecular function*, i.e., the biochemical activity (or potential activity) of a gene product; and *cellular component*, i.e., where in the cell that a gene product is localized and active.

Gene sequencing, comparative genomics, and gene annotation are symbiotic because all three activities help define genome function; improvements to these methods drive better annotations. For example, in the early days of the yeast genome sequencing project, annotation suffered from false positives as well as missed genes. The lack of other sequences for comparison also stymied annotation of conserved, noncoding sequences. New technologies such as ChIP-seq, nucleosome mapping, and proximity techniques were crucial for each genome revision. Indeed, while the yeast genome is arguably quite stable at the molecular level, it has undergone continuous revision, including changes in absolute gene number [28], with much of the reduction (nearly 10%) of the original ~ 6200 ORFs arising from comparative genomics. On the other hand, newly defined genes have been dominated by small ORFs that were not originally included because they did not pass the 100 amino acid threshold for being annotated as ORFs [29]. While the sequence of the yeast reference genome is arguably complete, the annotation of its gene complement will be continually revised as new technologies and insights are introduced.

## Yeast functional genomics

### Early efforts in functional genomics

Once the first phase of the yeast sequencing project was completed in 1996, the challenging task of assigning functions remained. Even before the yeast sequencing project was finished, several laboratories had constructed large-scale yeast mutant collections. For example, transposon tagging was used to generate 11,000 mutants in 2000 genes to track gene expression, protein localization, and disruption phenotypes [30]. The data from screens of 8000 strains performed in 20 different growth conditions were made widely available. This study highlighted the importance of making screening data publicly available [31] and helped lay the foundation for future genome-wide approaches to identify functionally related genes (for details, see reference [32]). These studies provided crucial early insights, including the observation that 20% of the genes are essential and further, which essentiality is condition-dependent. Analysis of the so-called nonessential genes argued against the idea that duplicated genes are redundant; indeed, experimental results showed that every gene, when deleted, exhibited a measurable fitness phenotype [33]. Such transposon-based screens have caveats—because insertions are not targeted, it is difficult to unambiguously distinguish between effects due to a gene disruption or a neighboring sequence feature. In addition, transposons have target sequence biases [34]. Nonetheless, these early studies underscored the need for a complete, systematic deletion collection that would encompass all essential and nonessential genes and simplify mutant interpretation by using complete, start-to-stop deletions.

### The S. cerevisiae deletion project

**Fig. 2:**
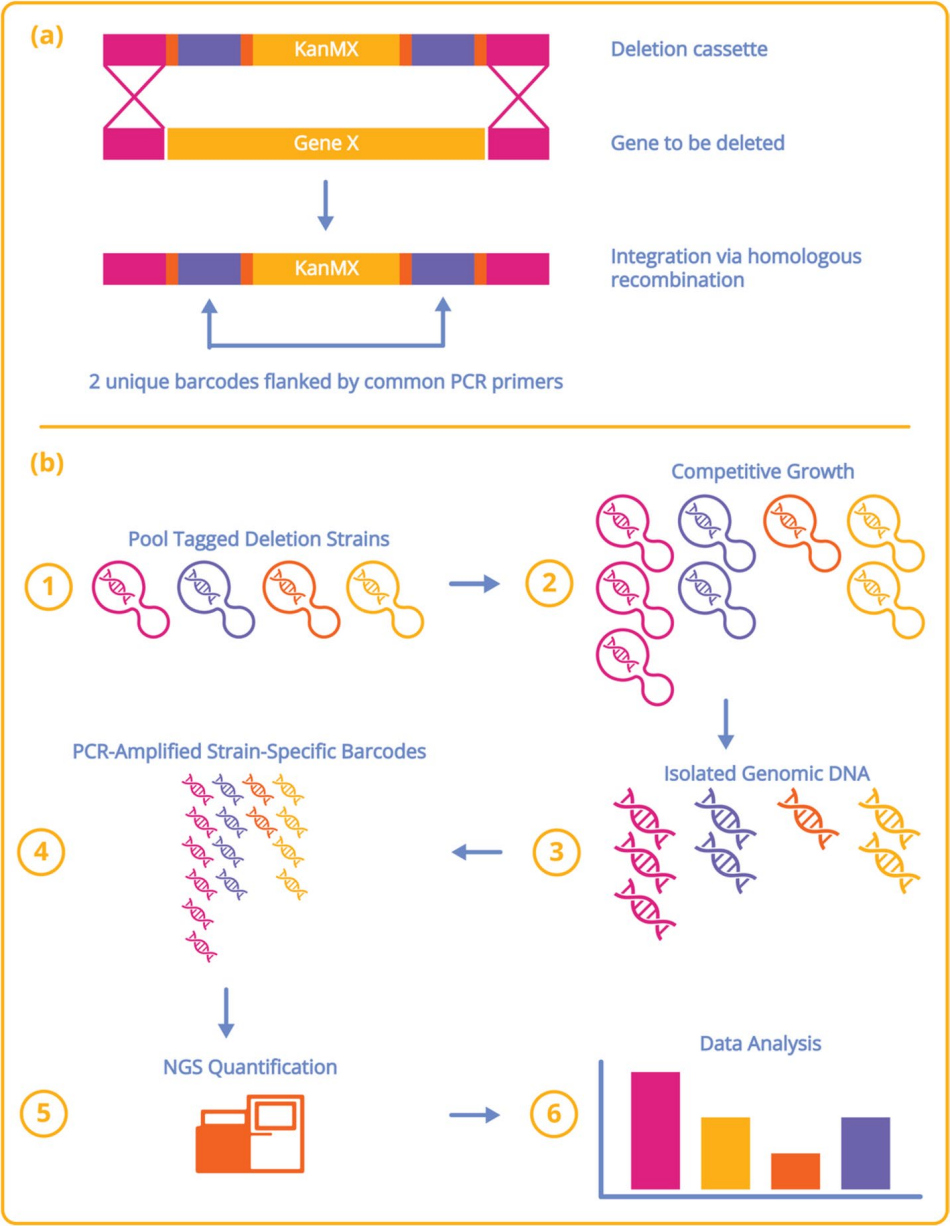
**a** Deletion strain strategy. The KanMX gene expressed from the *TEF1* promoter confers dominant selection of geneticin (G418) to yeast and was amplified with primers adding common primers (orange), molecular barcodes or tags (violet), and homologies with the chromosome (maroon). When transformed with the linear PCR product, cells replace the targeted ORF with the KanMX cassette flanked with UPtag and DOWNtag using homologous recombination. **b** Description of the competitive growth assay. Fitness profiling of pooled deletion strains involves six main steps. (1) Strains are first pooled at approximately equal abundance. (2) The pool is grown competitively in the condition of choice. If a gene is required for growth under this condition, the strain carrying this deletion will grow more slowly and become underrepresented in the culture (orange strain) over time. Similarly, resistant strains will grow faster and become overrepresented (maroon strain). (3) Genomic DNA is isolated from cells is harvested at the end of pooled growth. (4) Barcodes are amplified from the genomic DNA with universal primers in two PCR reactions, one for the uptags and one for the downtags. (5) Resulting PCR products are sequenced by NGS to quantify the tag sequences and, therefore, the relative abundance of each strain. (6) Tag intensities for the treatment sample are compared to tag intensities for a control sample to determine the relative fitness of each strain

The yeast *S. cerevisiae* deletion project (aka the yeast knockout or YKO collection) involved an international consortium of 16 laboratories (many of whom participated in the genome sequencing project) that, over the course of 3 years, deleted and distributed a systematic set of yeast deletion strains [35–37]. The history of this project and the essential roles of Ron Davis at Stanford and Mark Johnston at Washington University in St. Louis is reviewed in [7]. Each gene was precisely deleted—from the start-to-stop codon (non-inclusive)—and replaced (using mitotic recombination) with the KanMX deletion “cassette” (Fig. 2a) [38]. The KanMX gene inserted into the deletion locus in each mutant is flanked by two strain-specific 20-nucleotide sequences that serve as molecular barcodes to uniquely identify each deletion mutant. For the majority of mutants, the cassette was introduced into a diploid strain to produce the heterozygous deletion strain, which was sporulated to generate the *MATa* and *MATα* haploid deletion strains, followed by mating of the two haploids to generate the homozygous deletion strain [35]. Mutants that could not be constructed in diploids were made directly in haploids. In total, four sets of deletions were produced, all genes as heterozygous diploids, homozygous diploids, and both a and α haploids. A snapshot of the key pages of the original yeast deletion project website has been restored at “http://chemogenomics.pharmacy.ubc.ca/GGCN_Lab/SGDP/”—hosted by our lab at the University of British Columbia.

The unique sequence tags (i.e., barcodes) linked to each gene deletion allow the strains to be analyzed in parallel in competitive fitness assays. In these pooled experiments, a mixed culture containing every deletion mutant is grown, samples are collected at several times during growth, and the molecular barcode tags are amplified from the genomic DNA by PCR using common primers that flank the unique barcodes. The abundance of each deletion strain is then determined by quantifying the molecular barcodes by next-generation sequencing. The greater the degree that a gene is required for growth, the more rapidly that strain (and its corresponding sequence tags) diminish in the culture. Thus, all genes required for growth can be identified and ranked in order of their relative contribution to fitness in a single experiment [36, 37]. Figure 2 illustrates the workflow of these fitness assays.

The first progress report of the YKO consortium appeared in Science in 1999 [37] when a third of the deletion strains had been constructed. Major findings included the following: (1) of 2026 ORFs deleted, 17% were essential, (2) only half of all ORFs were previously known, and (3) in a competitive fitness assay across ~ 60 generations (performed in either minimal or rich medium using a pool comprised of 558 homozygous deletions), a fitness defect was revealed for 40% of the strains. The second consortium report, published in Nature in 2002 [36], announced the completion of the YKO collection and reported a tally of 18.7% essential genes out of 5916 ORFs. This landmark paper included full genome functional profiling of the homozygous deletion collection in five environmental stress conditions and in the antifungal drug Nystatin. Notable findings included a slow-growth phenotype for 15% of the strains in rich media as well as phenotypes for all mutants in well-characterized stress conditions, including high salinity (1-M NaCl) and high osmolarity (1.5-M sorbitol).

The 2002 study also established that there is *no correlation between the genes necessary for survival in a specific condition and those genes whose transcription is increased after exposure to that condition (for example, high salinity)*. [36]. This surprising lack of correlation between fitness and gene expression changes has subsequently been supported by numerous studies, including a report aptly titled “Transcriptional response of *S. cerevisiae* to DNA-damaging agents does not identify the genes that protect against these agents” [39]. Since this time, the YKO has been used to study the nuances of the stress response. For example, it has been suggested that the immediate mRNA stress response may be more important for surviving the next encounter with stress. A hallmark of the stress is now known to include a response at the protein level, which occurs much more rapidly than the transcriptional response and can include posttranscriptional events that provide “just in time production of genes” such as rapid reprogramming of translation through a variety of mechanisms—uORFs [40], stress granules [41], and active blocking of the exit of ribosomal subunits through the nuclear pore [42] to name a few.

### Derivative libraries and methodologies inspired by the YKO project

The completion of the YKO collection inspired the development and application of derivative strain collections (Table 2) and new genome-wide technologies. For example, genome-wide yeast libraries that reference the original YKO seminal publications have themselves been cited > 6000 times, including the yeast tandem affinity purification (TAP-tagged) collection [8], the GFP collection [43], and the glutathione-S-transferase (GST-tagged) ORF collection [44]. Other highly cited papers inspired by the YKO include novel methods for mutant construction in other organisms (for example, the *Arabidopsis thaliana* [45] and *E. coli* mutant collections [46]). Applications that leveraged the YKO project include genome-scale protein-complex analysis by mass spectrometry [47–49], protein microarrays [50], whole-genome analysis of synthetic genetic arrays (SGA) [9, 51, 52], and large-scale gene expression studies [32, 39, 53]. Although a detailed description of these libraries and technologies is beyond the scope of this review, we highlight several studies that have contributed to the understanding of yeast gene function and cellular processes.

**Table 2:** A selection of genome-wide yeast strain collections

Strain ollections	Supplier	Application	Reference
*HA-tagged proteins*	Horizon Discovery	Protein localization & purification	[30]
*Insertional mutant collection*	Horizon Discovery	Deletion studies	[30]
*GST-tagged ORFs*	Horizon Discovery	Ooverexpression screening	[50]
*Knockout collection*	Horizon Discovery	Deletion studies	[36]
*GFP-tagged ORFs*	Horizon Discovery	Protein localization	[43]
*TAP-tagged ORF collection*	Horizon Discovery	Protein quantification & purification	[8]
*Tet-promoters Hughes collection*	Horizon Discovery	Study of essential genes	[54]
*DAmP collection*	Horizon Discovery	Study of essential genes	[55]
*Yeast ORF collection*	Horizon Discovery	High-throughput chemical & genetic screens	[44]
*Yeast barcoder collection*	Horizon Discovery	Tool for barcoding collections	[56]
*Genomic tiling collection*	Horizon Discovery	Overexpression studies	[57]
*YFP fusion kinase collection*	Horizon Discovery	Localization and pathway biology	[58]
*Molecular barcoded yeast (MoBY) ORF*	Horizon Discovery	Drug-target screening	[59]
*ts collection essentials*	Euroscarf	Study of essential genes	[60]

Methods to generate synthetic genetic double mutants include the aforementioned synthetic genetic array (SGA) [61] and diploid-based synthetic lethality analysis on microarrays (dSLAM) [62]. The first SGA genetic interaction study described the systematic construction of all pairwise double mutants of dozens of haploid deletion strains [51, 61]. Subsequent, and ever-larger genetic interaction maps iterated this approach to produce a nearly complete dataset [52, 63, 64], including essential genes as ts (temperature-sensitive) or DAmP (decreased abundance by mRNA perturbation) hypomorphic alleles [65].

The TAP-tagged library [8] allowed the expression levels of all proteins in the cell to be quantified, while the *GFP* library [43] provided localization data on the proteome; together, these two libraries allowed an integrated view of the localization and abundance of nearly all proteins in the cell. The TAP-tagged library became instrumental in the first mass spectrometry-based genome-scale isolation of protein complexes. Subsequent improvements on these two collections have refined these datasets. For example, the SWAp-Tag (SWAT) was introduced by Weill et al. as a flexible library that facilitates the rapid construction of an endless number of variants [66] to characterize the yeast proteome for protein abundance, localization, topology, and interactions [67].

### Genome-wide phenotypic screens

The YKO collection has been used in thousands of genome-wide phenotypic assays and has provided insights into biological function, the response to stress, and the mechanism of drug action. Many genome-wide phenotypic screens have been independently repeated, with DNA metabolism and repair screens being prominent examples. The reader is referred to comprehensive phenotypic screening studies for details, e.g. [68–71]; here, we provide an overview of yeast phenotypic screens and highlight select examples.

In 2005, a screen of the heterozygous deletion collection revealed that 3% of ~ 5900 genes are haploinsufficient, manifesting a fitness defect in rich media [72]. At the time, there were two prevailing hypotheses of haploinsufficiency. The “balance hypothesis” posited that haploinsufficiency is due to a disruption in the stoichiometry of protein complex members [73], making the testable prediction that the haploinsufficient phenotype will be the same as the overexpression phenotype, because both scenarios disrupt the balance of protein subunits. Under this scenario, haploinsufficiency should be maintained regardless of the growth conditions because the stoichiometry of a protein complex would still be unbalanced. Alternatively, the “insufficient amounts hypothesis” postulated that haploinsufficiency results from reduced levels of protein product, rendering the cell less fit [72]. Deutchbauer observed that haploinsufficiency in minimal media was associated with a repression of gene expression, in contrast to predictions of the balance hypothesis—suggesting the importance of absolute transcript levels and indicating that specific gene products play a crucial role in growth limitation only in rich media [72]. Further, overexpression of 13 haploinsufficient genes did not cause a growth defect, and growth in minimal media (which slows the rate of cell division) alleviates most haploinsufficiency, as does any treatment that slows growth (e.g., high pH or growth inhibitors). Taken together, this work suggests that most cases of haploinsufficiency in yeast are caused by insufficient amounts of protein, with some exceptions like cytoskeletal genes (*ACT1*, *TUB1*, and *SPC97*) that maintain haploinsufficiency in both YPD and minimal media; for those genes, the balance hypothesis best explains their haploinsufficiency [72].

The majority of haploinsufficient genes have human homologs (107 of the 184), and all complexes that are haploinsufficient in yeast are present in humans. Of the 3% haploid-insufficient strains, over half were functionally related or functionally enriched for ribosomal function. The importance of ribosomal haploinsufficiency in eukaryotes is illustrated in *Drosophila* by the minute mutants that have several developmental abnormalities (for review, see [74]). In mammals, pathogenic effects of ribosome haploinsufficiency include Diamond-Blackfan anemia and 5 q- syndrome (a hematological disorder) [75]; also, haploinsufficiency of *RPL5* (a ribosomal 60S subunit) in human breast cancer cells accelerates tumor progression in a mouse model [76]. As the relevance of haploinsufficiency to human diseases and cancers becomes better characterized [77], the list of yeast haploinsufficient genes may serve as a valuable reference for understanding the role of their human orthologs in disease.

### Mitochondrial respiration screens

Many of the earliest studies on yeast, spearheaded by European brewers (e.g., the Carlsberg laboratories), focused on their ability to grow either in a fermentative manner or via respiration. Assessing the requirement of a gene for mitochondrial respiration is straightforward in yeast—the inability to grow on an obligate respiratory carbon source strongly implicates that the deleted gene product is required for this process. In 2002, Steinmetz et al. performed a systematic screen with varying carbon sources on the nonessential diploid deletion set and identified 466 genes whose deletion impaired mitochondrial respiration, including 265 that were novel [78]. Three independent colony-based, genome-wide studies also screened the deletion collection for genes required for respiratory growth [79–81]. As opposed to liquid growth assays that typically measure fitness by light scattering, colony-based yeast studies measure fitness based on colony size. In one of the most recent of these studies, Merz and Westermann included a welcome comparison of these results, revealing an overlap of 176 genes between all three colony-based studies, each representing approximately half of the genes identified in each individual screen [81]. The discrepancy between the number of respiratory-deficient mutants identified between studies could come from several sources, but regardless of the cause, this observation highlights a limitation of the YKO, namely, it is limited to a single genetic background. Newer tools (discussed below) should expand the deletion approach to other strains—indeed, several small-scale efforts have shown the utility of this approach in the context of wine strains and those that undergo pseudohyphal growth [82, 83].

### Caveats on the yeast deletion collection

Despite its merits, the YKO has several key limitations that limit its utility. These include the fact that the YKO represents a single genetic background, which contains several well-characterized polymorphisms that compromise sporulation, mitochondrial function, and other less obvious phenotypes [84]. While there are other yeast deletion mutant collections, most of them are hybrids in which the deletion cassettes were derived from the original YKO (for example, [83] and [85]). Additionally, improper maintenance of large strain collections (i.e., wrong colony on plate or polyclonal colonies) can lead to the wrong mutant being tested. Another confounding factor is that individual mutant strains may have acquired mutations (e.g., second-site suppressors, aneuploidy, and diploidization). Accordingly, it is recommended that results from YKO experiments (either individually or in pools) should be independently constructed and validated. The compact nature of the yeast genome often complicates the study of individual genes. In other words, deletion of one gene can occasionally disrupt the promoter, terminator, or coding sequences of nearby genes on the opposite strand of genomic DNA. Interestingly, very closely spaced deletion mutant pairs can occasionally be used to confirm neighboring gene function as described below for *SIR2*. Despite these caveats, the broad uptake of the YKO as an experimental platform is clear—in 2023, Anastasia Baryshnikova’s group analyzed ~ 14,500 yeast knockout screens and clustered these datasets into what they dubbed the Yeast Phenome, illustrating the continued usefulness of the YKO [86].

## Protein–protein interactions

### Two-hybrid studies

One of the first methods to study protein–protein interactions (PPIs) at scale was the two-hybrid system. In the original version of the assay, two query proteins are constructed, with the “bait” protein fused to the DNA-binding domain of the Gal4 protein and the “prey” protein fused to the activation domain of Gal4. If these two proteins physically interact, Gal4 activity is restored and can be measured via activation of a reporter gene. An alternative assay, called “the interaction trap,” was introduced by Golemis et al. in 2008 [87]. By generating genome-wide “orfeome” collections to be used as either bait or prey libraries, these assays can be carried out on a genome-wide scale in an array format (reviewed in reference [88]). In two early extensive two-hybrid studies, all possible combinations of ~ 6000 proteins in yeast were interrogated. One study identified 841 interactions [89], and the other identified 691 [90]. These two reports made progress towards a comprehensive protein–protein interaction map, yet their datasets shared only 141 genes in common, 40 of which were known interactions. Technical differences, such as different reporter plasmids, may explain the lack of agreement, and additional limitations of the two-hybrid system should be considered. For example, when fused to the *Gal4* binding or activation domain, many proteins may fail to fold properly, contributing to a false-positive rate of approximately 25% per unique interaction for yeast [91]. With the introduction of NGS, 2-hybrid screens have been adapted to massively parallel formats; nonetheless, careful validation of any hits is still required. Newer yeast protein–protein interaction technologies include those from the David Baker’s lab at the University of Washington [92], companies (e.g., *A-Alpha Bio*), as well as PROPER-seq, a technique which infers PPIs based on assessing the transcriptome [93].

### Protein complexes identified used mass spectrometry

Comprehensive protein–protein interaction maps, powered by developments of tagged proteomes and the development of unbiased mass spectrometry methodologies, have increased the breadth and depth of our understanding of the yeast interactome. The TAP (tandem affinity purification) method is used to purify TAP-tagged proteins and their associated proteins. The TAP tag comprises a calmodulin-binding peptide (CPB), a protein A moiety, and a tobacco etch virus (TEV) protease cleavage to facilitate the isolation process (see reference [8]). Two early large-scale mass spectrometry studies took advantage of the TAP tag fusion collection to identify all protein complexes in the yeast genome. Gavin et al. identified 491 complexes comprising 23% of the yeast proteome (257 of these complexes were novel). Many of the proteins identified are “modular” with some always appearing together, while others present in more than one complex [49]. Krogan et al. identified 547 complexes, comprising 47% of the yeast proteome, with 2702 proteins in total [48]. It is difficult to compare the two datasets because they used different methods, and indeed, only six complexes were identical between the two sets. Hart et al. [94] integrated the two datasets along with a third dataset (from Ho et al., [47]) and found a consensus of 1689 proteins representing 390 protein complexes. Of the 132 with 4 or more subunits, 69% are highly enriched for specific GO component annotations suggesting that the complexes are highly accurate. Essential genes are enriched in complexes and based on the high proportion of complexes that are already annotated, and the relative dearth of uncharacterized genes in the high confidence data suggests that these studies may have largely saturated the fraction of the yeast “complexome” that is accessible in these conditions using these methods of isolation [95, 96].

## Chemogenomics: identifying drug targets

Chemogenomic profiling is a method designed to study the genome-wide response to small molecules. The ability to identify drug targets in vivo in an unbiased manner without prior knowledge has made yeast instrumental in such mechanism-of-action studies. Traditional chemogenomic approaches to determine the mechanism of action (MoA) of drugs include isolation of drug-resistant mutants followed by genetic mapping. While this mutational approach can identify the drug target, it is difficult to scale. Alternatively, one can clone a drug target by complementation [97]. In one early cloning-by-complementation study, a strain mutated for the gene encoding a drug target (HMG-CoA reductase) was transformed with a genomic DNA clone bank to identify drug-resistant colonies able to grow on solid media containing lovastatin [98]. This method inspired multicopy suppression profiling (MSP), where a library of clones is introduced, in parallel, into a pool of mutants, and once resistant strains are identified, the complementing plasmid-borne gene is sequenced to reveal candidate drug targets. Traditional MSP screens involve plating techniques and characterization of individual clones by sequencing [99]. They are prone to false negatives, for example, if the wrong time point is assayed or the wrong drug concentration is used, and the results can be dominated by a gene product unrelated to the drug target. There are now several well-characterized overexpression libraries that can be used for high-throughput studies [57, 100], including the molecular-barcoded yeast ORF (MoBY-ORF) collection built by Ho et al. in 2009 [59]. This collection is barcoded, and because each CEN-based plasmid carries a single ORF flanked by its native upstream and downstream genomic sequences, the copy number is low and predictable, minimizing overexpression toxicity [101]. Indeed, high-level overexpression can disrupt cellular homeostasis, and several groups have exploited this phenotype to find inhibitors that alleviate the fitness defect caused by overexpression of toxic proteins [102–104].

Haploinsufficiency profiling–homozygous profiling (HIP − HOP) is a gene-dose assay that relies on an increase in drug sensitivity to identify drug targets. The HIP assay relies on the drug-induced haploinsufficiency phenotype, which is based on the observation that reducing the copy number of a drug target from two copies to one copy in diploid yeast results in increased sensitivity to a compound that inhibits the gene product of the heterozygous locus [105]. HIP uses essential heterozygous deletion strains in competitive fitness assays combined with quantitative analysis of the molecular barcodes to identify relative strain abundance—strains most sensitive to the drug provide a ranked list of the most likely drug target candidates [10, 105–107]. HIP has the advantage of simultaneously identifying both the inhibitory compound and its candidate target(s) without prior knowledge of either. In some cases, a 50% decrease in gene dosage is not sufficient to identify the drug target. In these cases, complementary approaches that use DAmP (decreased abundance by mRNA perturbation) alleles can be used [65]. The DAmP collection is a set of hypomorphic alleles that carry a disruption in the 3′-untranslated region in the essential genes, which destabilizes the corresponding RNA transcript and results in a ~ 5–50-fold decrease in mRNA levels [65].

The HOP assay is analogous to HIP, except that the homozygous deletion collection is used. It complements the HIP assay by providing a ranked list of genes (by virtue of their deletion strain sensitivity) that buffer the target pathway, including those that comprise pathway components as well as genes involved in multidrug resistance (e.g., drug transport, detoxification, and metabolism). When combined, HIP–HOP chemogenomic profiles give a comprehensive view of drug mechanism along with primary and secondary targets, identifying all genes required for drug exposure–response in a single assay. HIP − HOP has been successfully used to identify the target of known and novel compounds [10, 106–109]. An illustrative example includes *Sir2*, a histone deacetylase, as the target of tenovin, a small-molecule p53 activator [110]. This study screened the heterozygous diploid deletion with a derivative of tenovin to show that *sir2* deletion strains manifested tenovin-induced haploinsufficiency. While none of the silent information regulator genes was represented in the YKO (because they are unable to mate), the deletion of an adjacent dubious ORF (YDL041W) removed the first 300 nucleotides of *SIR2*, abolishing its function and establishing Sir2 as a potential tenovin target.

**Fig. 3:**
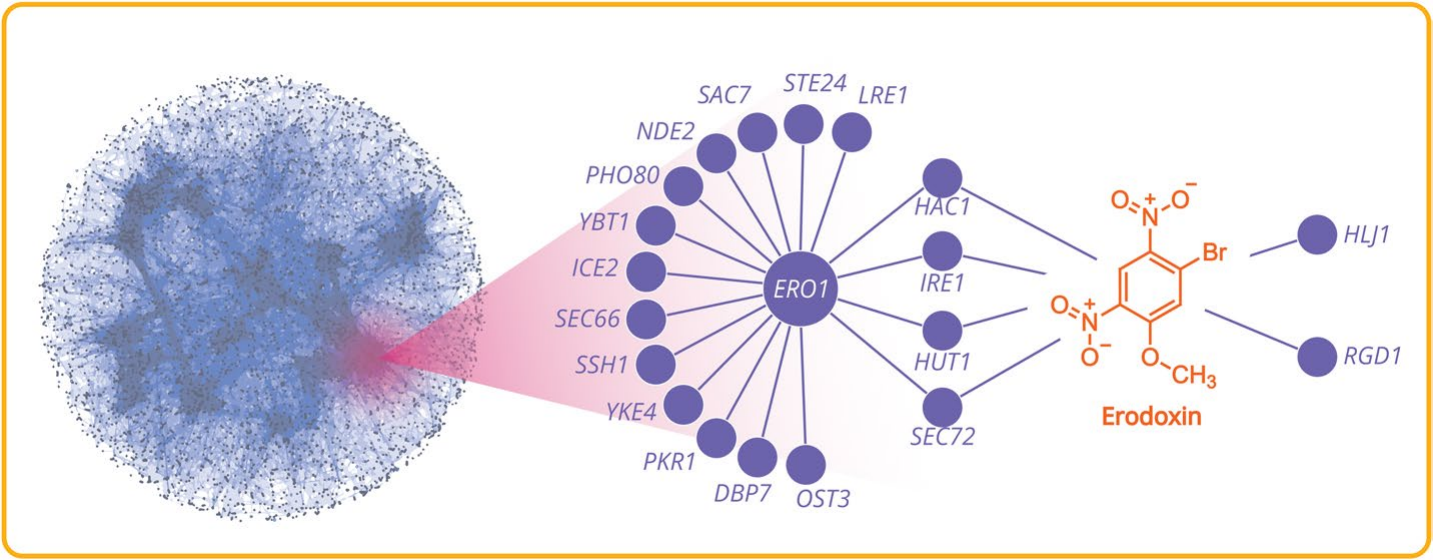
Left: An overview of genetic and chemical–genetic interactions in yeast compiled from diverse large-scale data sources, including SGA, PPI, and chemogenomics (left panel). The right panel shows a higher resolution representation of gene–gene and gene–drug interactions as they relate to diverse cellular processes. Genes are visualized as circular nodes, while the interactions between them are depicted as lines connecting these nodes. It shows the genetic interactions for the ERO1 module comprising a network of genes with shared genetic interactions, some of which are shared by the chemical–genetic interaction network induced by treatment with Erodoxin, a small molecular Ero1 inhibitor whose structure is illustrated

Combining the results of several thousand such HIP − HOP screens revealed that the cellular response to small molecules is limited and can be described by 45 “signature” chemical-genetic interaction profiles that are detectable in other large-scale genomic datasets, suggesting that they represent fundamental, conserved small-molecule response systems present across eukaryotic cells [10]. Figure 3 shows an overview of a HIP − HOP growth assay result, representing chemical–genetic interactions of a small molecule, erodoxin, with a complex network of yeast genes highly enriched for “post-translational protein targeting to membrane” and “endoplasmic reticulum membrane” genes.

Other genome-wide chemogenomic strategies (e.g., SGA and gene–expression profiling) rely on “guilt by association” to identify the target of a drug from a compendium of reference profiles (e.g. genetic interactions or gene expression) [111–114]. Since drugs with similar mechanisms have similar chemical–genetic profiles, the drug target can be inferred by global analysis of chemical–genetic profiles to uncover reference compounds with established MoA [113].

### Recent advances in chemogenomics

With advancements in computational and statistical techniques, combined with the increasing ease of genetic engineering, functional genomic studies in yeast have flourished. Machine learning approaches have been successfully used in yeast functional genomics, including cell growth prediction, pathway engineering, and chemogenomics [115–117]. Recently, genome-wide CRISPR screens (including CRIPSRi) have been applied to yeast, and to date (see below), the results are in agreement with more traditional molecular techniques.

The Charlie Boone/Brenda Andrews laboratories have extensively surveyed gene–gene interactions across the genome. In 2020, they extended their pioneering digenic interaction studies to interrogate trigenic interactions to identify those genes that have maintained their functional overlap versus those that have evolved novel functions [118]. A large number (~ 550,000 double and ~ 260,000 triple mutants) were screened; ~ 4700 negative digenic interactions and ~ 2500 negative trigenic interactions were identified. Statistical analysis suggested that two-thirds of paralogs have functionally diverged during the course of evolution, while one-third are functionally redundant [118].

Recently, researchers have used genome-scale CRISPR screens in yeast to both improve the technology and to apply it in novel ways. Smith et al. performed a genome-wide CRISPR interference screen to investigate the effectiveness of gRNA for transcriptional repression [119]. Using an inducible, plasmid-based CRISPRi system (with 20 gRNAs directed to 20 genes whose expression should influence sensitivity to specific small molecules) along with 18 small molecules, the effects of gRNAs on CRIPSRi-induced fitness defects were studied, and generalizable characteristics associated with gRNA efficacy were assessed. The chemical–genetic interactions identified by this strategy were precisely consistent with previously described interactions. More recently, Momen-Roknabadi et al. validated the CRISPR approach by introducing a genome-scale, inducible CRISPRi library, which they applied to uncover haploinsufficient genes and enzymatic and regulatory genes involved in adenine and arginine biosynthesis [120].

For yeast CRISPR applications, targeting genes and their regulatory elements is straightforward, but modifying SNPs is more difficult, owing to the high degree of sequence similarity between the guide and the donor (which can result in loss of the variant through cell death or mutation by NHEJ) and uncertainty about the availability of PAMs near the SNP. To address these limitations, Lars Steinmetz’s lab published a CRISPR–Cas9-based method called MAGESTIC (*m*ultiplexed *a*ccurate genome *e*diting with *s*hort, *t*rackable, *i*ntegrated *c*ellular barcodes) for variant analysis [121]. Using MAGESTIC, they carried out a saturation mutagenesis experiment on the essential gene *SEC14* and determined which amino acids are crucial for chemical inhibition of lipid signaling. They showed that the editing efficacy can be improved five-fold when the donor DNA is recruited to the site of breaks using LexA–*Fkh1p* fusion protein [121]. Most genome-wide genotype–phenotype screens had been restricted to a single mode of alteration—deletion, repression, or overexpression. Taking advantage of the trifunctional CRISPR system (aka CRISPR-AID [122]), Huimin Zhao’s lab developed multifunctional genome-wide CRISPR (MAGIC) to regulate the expression level of each gene to prespecified levels. They constructed three genome-scale gRNA-expressing plasmid libraries for upregulating, downregulating, and deleting genes, representing new options for yeast functional libraries [123].

In 2021, Alford et al. introduced a reverse genetic method called ReporterSeq to define pathways involved in the yeast stress response. ReporterSeq identifies genes that regulate stress-induced transcription factors in a time-resolved manner in different environments by pairing the enumeration of RNA-encoded barcodes to pathway-specific outputs that are enumerated by DNA sequencing. Employing ReporterSeq in 15 stress environments, they discovered novel, stress-specific, time-specific, and constitutive regulators and suggest that this method could be applied with any encodable genetic perturbation (e.g., RNAi, CRISPR knockouts) [124].

**Fig. 4:**
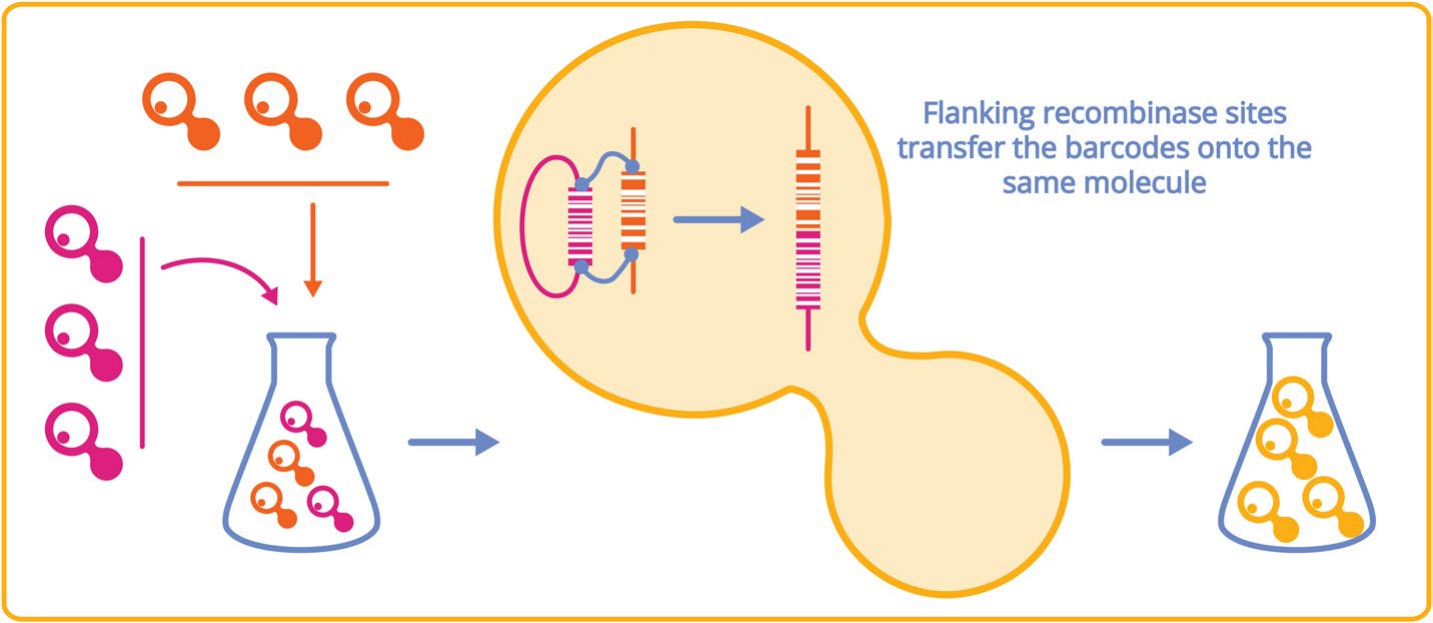
The barcode fusion genetics (BFG) approach combines two barcoded populations into individual cells to increase screening throughput [127]. The BFG technology enables phenotypic analysis of a heterogeneous pool of millions of yeast strains, each having two engineered loci or genes of interest. In BFG, a doubly engineered cell pool is prepared using either (1) mating of two haploid populations (maroon and orange strains in the left panel), (2) plasmid transformation with a barcoded gene bank, or (3) microencapsulation of two yeast cells into a single emulsion droplet. Once segregated, the transfer of a DNA barcode flanked by site-specific recombination (based upon Cre-Lox recombination) sequences is initiated to yield a single, doubly barcoded molecule (center panel). Strain abundances can then be quantified by amplification and deep sequencing of fused barcodes (right panel)

The utility of the genetically encoded barcodes of the YKO has recently been extended by several groups that combined pooled approaches to genetic interaction mapping with the ability to generate “barcode fusions” between two distinct cells. In brief, these approaches rely on the ability to isolate interacting cells, either by (1) mating, (2) by transforming cells carrying one barcoded locus with a plasmid containing a second barcode, or (3) by encapsulating cells within oil-in-water emulsions. Cells or pairs of cells are then subjected to barcode fusion either by Cre-Lox recombination or fusion PCR, followed by massively parallel sequencing [125–127] as shown in Fig. 4. Barcode-fusion genetics and variations on this theme promise massive increase in both scope and scale.

## Bioinformatics

The Yeast Genome Project established the field of functional genomics in the twenty-first century. Concurrently, digital computers and broad accessibility to the Internet made reconstructing genomes from sequence fragments a reality. Bioinformatics played a vital role in the interpretation of the information encoded in the yeast genome. The computational transformation of primary sequence data into biologically relevant information was first reported on a large scale by Frishman and team in 2001; they published the first systematic genome analysis pipeline based on their experience with yeast [128]. Soon after, databases such as *Saccharomyces* Genome Database (SGD) [25], Comprehensive Yeast Genome Database (CYGD) [129], *YeastWeb* [129], and others were built to store the raw sequences and bioinformatically refined data. Today, SGD has become a gold-standard resource for genetics and molecular biology of yeast *S. cerevisiae*. It also provides detailed information about genes and their biological functions, as well as resources and tools for exploring sequence data [130]. SGD has spawned dozens of organism-specific databases with similar aims. While the SGD has evolved into a premier model organism database, BioGRID (Biological General Repository for Interaction Datasets) [131], originally launched in 2006 to comprehensively curate all available biological interaction data generated in yeast, has expanded to encompass other organisms, including human cells. This open-access database resource is highly complementary to SGD, with manually curated protein and genetic interactions from multiple species. To date, BioGRID curators have read more than 197,000 publications, a number which should increase greatly with the broader adoption of large language models or LLMs (see reference [132] for a prescient review of this topic).

To accommodate the increasingly complex and diverse needs of researchers for searching and comparing data, SGD introduced *YeastMine* [133], a multifaceted search and retrieval environment that provides access to diverse data types. This tool is functionally integrated into *Galaxy* [134], an interactive system that combines the power of existing genome annotation databases with a web portal to enable researchers to search remote resources, combine data from independent queries, and visualize the results. Increasingly, these research tools are being made available from strain and plasmid repositories such as the well-established American Type Culture Collection (ATCC) and newer sources such as Addgene. Also, bioinformatics and data visualization tools like *TheCellMap* [135] and discipline-focused databases (such as those curated by in the Nucleic Acids Research annual database issue) add to the growing bioinformatics toolkit.

The open-source software project *Cytoscape*, which integrates biomolecular interaction networks with high-throughput expression data and other molecular states into a unified conceptual framework [136], has been instrumental in yeast bioinformatics, especially when used in conjunction with large databases of protein–protein, protein-DNA, and genetic interactions. Cytoscape possesses functionalities to query interaction networks, visually integrate them with expression profiles, phenotypes, and other molecular states, and it can link to databases of functional annotations. Another Cytoscape-based app is *GeneMANIA*, a web-based tool that helps predict the function of genes and gene sets using a very large set of functional association data [137]; this data includes protein and genetic interactions, pathways, co-expression, co-localization, and protein domain similarity. *STRING* is another well-known web tool providing orthology prediction, functional and physical network prediction, etc. A similar web tool with additional capabilities to use transcriptional regulation association and mutant phenotype association from yeast has been developed, called *YAGM* (Yeast Associated Genes Miner) [138].

Whole-genome sequencing, coupled with bioinformatics, has enabled fast and cost-effective mutation identification. Multiple web-based tools developed by the Fritz Roth lab, such as *ChromoZoom* [139] and *FuncBase* [140], have been developed. ChromoZoom is a genome browser that hosts tracks for yeast and human genomes, whereas FuncBase enables browsing of quantitative gene function assignments for yeast, mouse, and human genes. In addition, the Roth lab has been instrumental in developing *MaveDB*, a central repository allowing researchers to store and publish processed multiplex assays of variant effect (MAVE) datasets, such as deep mutational scans and massively parallel reporter assays [141]. This work has been done in collaboration with Douglas Fowler’s lab—who has contributed to developing genome engineering tools and combining cutting-edge genomic methods with computational analyses to measure the consequences of tens of thousands of DNA sequence alterations simultaneously.

To identify new yeast mutants, *Mudi or Mutation discovery* is a browser-accessible and easy-to-use bioinformatics tool that enables “one-click” identification of causative mutations from sequence data [142]. *CRIMEtoYHU* (CTY) is a similar web tool that helps geneticists evaluate the functional impact of cancer-associated missense variants. Since *S. cerevisiae* and humans share thousands of protein-coding genes, yeast humanization is useful for deciphering the functional consequences of human genetic variants found, for example, in cancer and providing information on the pathogenicity of missense variants (see below). CTY finds yeast homologous genes, identifies the corresponding variants, and simultaneously determines the transferability of human variants to their yeast counterparts by assigning a reliability score which may serve as a predictor for the validity of a functional assay. It analyzes and ranks newly identified mutations or mutations from the *COSMIC database*. Then, it provides information about the functional conservation between yeast and human and shows the mutation distribution in human genes [143].

## Humanization of yeast

Functional genomics can be used to further many aims, such as providing a comprehensive description of the overall functioning of a single-cell organism at the systems level. Additionally, by virtue of the evolutionary conservation between yeast and human cells, we may better understand human physiology and pathophysiology. A complementary approach marries these two aims by directly engineering yeast to express human proteins of interest. Specifically, this work involves identifying the human orthologs of yeast genes for expression in yeast. By Eugene Koonin’s definition, “Orthologs are genes originating from a single ancestral gene in the last common ancestor of the compared genomes” [144]. The ortholog–function hypothesis states that orthologous genes have identical or similar functions in divergent species [145]. By exchanging orthologs from one species into another, many individual studies have directly investigated the conservation of function. When direct investigation of human biology is constrained by ethical and practical concerns, model organisms can be useful proxies. For example, *S. cerevisiae* has proven to be a vital tool for deciphering much of the biology that underpins human cell function and disease. In 1985, for instance, Kataoka et al. showed that yeast cells deleted for RAS genes cannot germinate, but that the expression of a chimeric mammalian/yeast RAS gene under control of a galactose-inducible promotor can complement this defect [146]. Humanized yeast cells can also be employed to investigate the function of human genes in response to drug treatment, including screening small molecules for their activity against human protein targets such as DNA damage checkpoint repair (*DDCR*) inhibitors [147].

Some of the most highly conserved genes in the human and yeast genomes encode proteins involved in cell machinery (e.g., DNA replication and repair) whose defects lead to various disorders and diseases. Such genes can display a range of genetic variation, which can be difficult to study in the original organism. Humanized yeast provides an in vivo platform for screening drugs or small molecules that inhibit human proteins [148]. For example, *FEN1* (human ortholog of *RAD27* in yeast) is a protein that functions in DNA replication and repair. The expression of Fen1 in many cancer types is very high, supporting the hyper-proliferation of cancer cells. Phil Hieter’s lab used this system to test two known human *FEN1* inhibitors: PTPD (a N-hydroxyurea-based compound) and a derivative of NSC-13755, while both compounds inhibited growth of a humanized *FEN1* yeast strain in the presence of MMS, only PTPD was a potent, specific inhibitor of *hFEN1*. In contrast. NSC-13755 was associated with general toxicity [149].

**Fig. 5:**
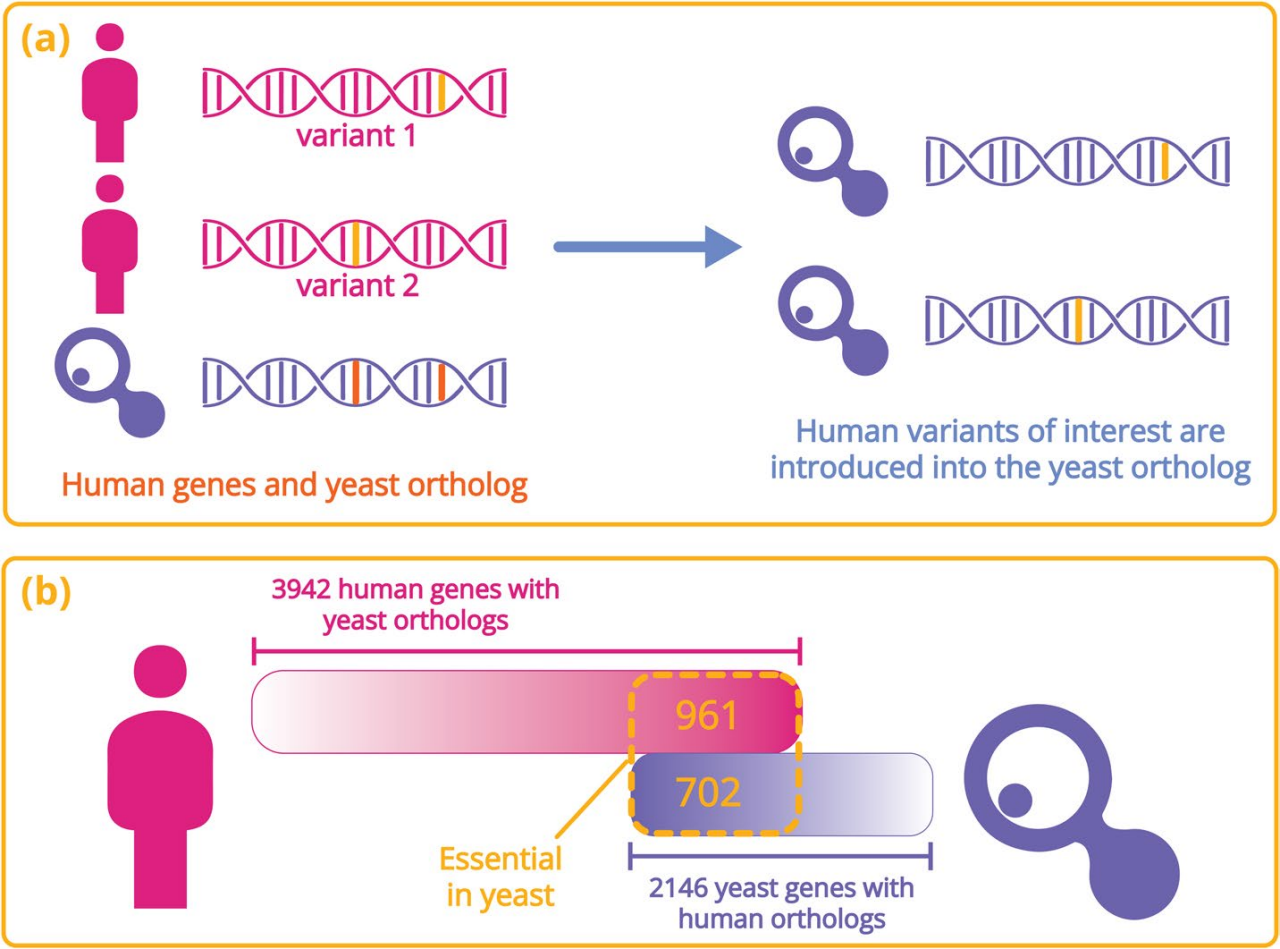
**a** Graphical representation of how the evolutionary conservation between orthologous human and yeast genes can be experimentally exploited to study human gene function in yeast. In this example, two variants in a human gene are shown, along with the engineered yeast ortholog (left panel). The human gene variants can be introduced, one at a time, and tested for diverse phenotypes (right panel). **b** A summary of the essential yeast genes for which experimental data exists for all 1:1 human orthologs

Since the 2010s, researchers have been engineering yeast with orthologous genes from plants, humans, and even prokaryotes to test their functional compatibility [150–154]. In a landmark study from Edward Marcotte’s laboratory, the “swappability” of 414 essential genes in yeast was tested by replacement with their human orthologs. Nearly half of the human orthologs (47%) could complement the yeast growth defect. The ability of many human genes to substitute for their yeast orthologs indicates the remarkable level of functional conservation in eukaryotic systems throughout billion-year evolutionary periods (Fig. 5). Conspicuously, this high degree of “swappability” was not highly correlated with sequence similarity; instead, genes involved in specific complexes or pathways behaved in the same way [151]. Fully humanized protein complexes may be restricted in their capacity to interact with their correct partners in the setting of a yeast cell, similar to particular humanized sites without the context of their human protein [6]. Indeed, the modular nature of replaceability suggests that this may be the case and hints that the inability to properly form the necessary interactions may be a driving force behind certain proteins being unable to replace their yeast counterparts [6].

An important humanization study was carried out in Jef Boeke’s lab where they coaxed yeast to survive solely with human core histones in their nucleosomes. They used a plasmid-shuffle strategy to replace yeast histones with human counterparts, resulting in a yeast cell with a humanized epigenome [155]. This work showed that the human core histones are able to function in *S. cerevisiae* (albeit with reduced fitness) without the accessory human genes to deposit them on to DNA. This work, while highlighting the power of yeast humanization, also underscores the challenges—when Truong and Boeke measured the growth rate of humanized yeast under the growth conditions of both yeast and human cells, they found that yeast bearing humanized nucleosomes required genomic suppressors to recover their growth rate [155]. In an interesting twist on complementing yeast mutants with a human ortholog, Sturley’s group has developed a yeast-human model of Niemann-Pick disease and showed that the yeast *NPC1* gene can functionally complement cells derived from NPC patients [156].

Numerous complex interactions with various organelles and signaling pathways that are present in human cells are absent in yeast. In these instances, model, humanized strains can be used to examine the pathway in a “clean” background. For example, Boonekamp et al. recently reported the first successful humanization of skeletal muscle glycolysis in yeast, opening up the possibility of exploring human glycolysis in yeast [139]. When paired with evolutionary strategies, single gene and complete pathway transplantation can demonstrate the extraordinary conservation of glycolytic and moonlighting functions as well as context-dependent responses. For instance, the study showed that human hexokinases 1 and 2, but not 4, required alterations in their catalytic or allosteric regions in order to function in yeast, while hexokinase 3 was unable to complement its yeast ortholog. Human glycolytic enzyme turnover rates were preserved in both yeast and human cell cultures when compared to human tissues. The construction of metazoan models that are tailored to certain species, tissues, and diseases is made possible by this example of the transplanting of a complete critical pathway [139]. However, the characterization of the physiological and cellular impact of the transplantation is one of these techniques’ general limitations.

The future of yeast humanization is promising. Perhaps the most exciting avenue for humanized yeast is the potential for constructing “personalized” strains, expressing any given allele of a human gene or combinations of genes, to make personalized yeast avatars. Finally, we need not restrict ourselves to replacing only orthologous genes. While likely not a widespread phenomenon, complementation of yeast mutants by non-orthologous human genes may prove useful, especially in cases where orthologs do not complement or where orthologs cannot be identified.

## Yeast as a synthetic biology innovator

Cameron et al. generally describe synthetic biology as the use of molecular biology tools and techniques to forward-engineer a desired function to produce a desired cellular behavior [157]. Although many microorganisms have been exploited using genome engineering to produce a specific cellular function, *E. coli* and *S. cerevisiae* are the preeminent test beds of synthetic biology, and they remain crucial drivers of the field. The history of synthetic biology is discussed in a detailed review by Cameron et al. [157], and here, we highlight the landmark events of the field, focusing on yeast contributions.

In the mid-2000s, a crucial milestone of metabolic engineering was published by Jay D. Keasling’s lab, in which they reported the biosynthesis of an antimalarial lactone, artemisinin, in yeast [158]. They also developed *E. coli* strains capable of producing any terpenoid compound for which a terpene synthase gene is available [159]. Such achievements paved the way for commercial and industrial applications of synthetic biology.

The pioneers of synthetic biology aimed for comprehensive control of cellular function, as envisioned at the SB1.0 conference. Venter and colleagues used new DNA assembly techniques to create a viable bacterial cell that was “rebooted” by a chemically synthesized genome. Subsequently, two teams leveraged CRISPR to minimize the number of chromosomes in haploid yeast cells from 16 to 1 or 2 (aka Sc2.0), and the results were published in the same issue of *Nature* in August 2018; Jef Boeke’s lab reported a synthetic yeast cell with only two chromosomes; however, fusing these two giant chromosomes was lethal to the cells [160]. In contrast, Shao et al. managed to engineer a functional yeast cell with a single chromosome [161]. Surprisingly, in both *n* = 1 and *n* = 2 strains, the expression of only a few genes was significantly different from wild type. Such efforts in synthetic biology will allow us to address very fundamental questions such as the following: (1) why almost all eukaryotes distribute their genome into multiple chromosomes, (2) if particular chromosome numbers can be of benefit for specific species, and (3) how chromosomal structures affect cell viability.

Seven years after the first laboratory-scale synthesis of artemisinin using yeast, Amyris, Inc. engineered an optimized artemisinin acid pathway in yeast, which led to the large-scale production of the drug [162]. As a result, hundreds of thousands of individuals in lower-income countries have access to antimalarial the drug at a low cost. Another event that has indeed revolutionized synthetic biology was the emergence of CRISPR-Cas technology, pioneered by Jennifer Doudna, Emmanuelle Charpentier, and others (for review, see reference [163]). In 2013, George Church’s team reported a CRISPR-based approach for site-specific mutagenesis and allelic replacement in yeast, which demonstrated that the introduction of targeted double-strand breaks significantly enhances the rates of homologous recombination. They reported a fivefold increase in recombination rates when using single-stranded oligonucleotide donors and a remarkable 130-fold increase when employing double-stranded oligonucleotide donors. This study laid the foundation for efficient site-specific mutagenesis and allelic replacement in yeast [164]. In an important proof-of-principle experiment, Christina Smolke’s lab reported the complete biosynthesis of opioids in engineered yeast cells using sugar as the starting material [165]. The resulting yeast cell factories were modified with more than 20 genes expressing enzymes from plants, mammals, bacteria, and yeast itself [165].

Because the S288c strain used in the Sc2.0 project lacks many of the genes that give industrial and environmental isolates their phenotypic variation, Kutyna et al. created a neo-chromosome that incorporates many different yeast pan-genomic components [166]. This “neo-existence” chromosome gives the Sc2.0 parental strain phenotypic plasticity, including an increase in the variety of usable carbon sources. The ability to adapt synthetic strains to a larger range of conditions may thus be made possible by the inclusion of this neo-chromosome within the Sc2.0 backbone. This process will be crucial to moving Sc2.0 from the lab into more practical industrial applications.

As we evolve from genomics as a “read-only” discipline (i.e., decoding genomes by sequencing) to a “read–write discipline” (combining sequencing with synthesis), yeast will remain a primary organism for the development of modular biofoundries for synthetic chemistry of diverse biomolecules, including human pharmaceuticals.

## Looking ahead

In 2011, Botstein and Fink published a compelling perspective entitled “Yeast: An Experimental Organism for 21st Century Biology” [167]—an update of the paper published by the same authors 23 years earlier [168]. They initially posited that yeast, owing to a convergence of genetics and molecular biology, was poised to become the premier experimental organism for modern biology. These predictions were prescient, and indeed, yeast has exceeded expectations, particularly with respect to being the chief innovator in the interfacial disciplines of functional genomics and systems biology. From our perch in 2023, we suggest that these new fields will expand in scale, scope, and impact. With regard to single-cell genomics, analysis of yeast represents a powerful means to understand both genetic and epigenetic contributions to cell variation. Finally, the extraordinary advances in yeast bioengineering, including a complete recoding of the genome, promise to bridge the gap between yeast as a living cell and a semisynthetic biosensor.

As has been true for the past 150 years, the impact of yeast on scientific research is vast and not completely predictable. Below, we highlight some future prospects for the technological and experimental development of yeast in the near future, with examples of each.

### Three areas to watch

**Fig. 6:**
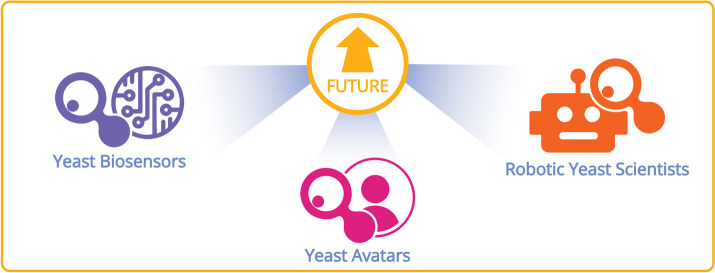
A view of a few near-term opportunities for which yeast research and biotechnology may have an impact. The arrow in the center highlights the subdisciplines of yeast biosensors for monitoring cellular activities, environmental changes, etc., yeast avatars as a means to study normal and pathological conserved gene function and the advent of Automated Yeast Science in which high-throughput technologies are married to machine learning in an iterative process

As yeast enters its second century as a model organism, it is fair to ask if its best days are in the rear-view mirror. The list of genetic and molecular features that were once exclusive to yeast experimentalists has undergone a transformation with the advent of NGS and high-performance computing. These technologies, which make any genotype accessible, have fueled the expansion of increasingly sophisticated genome modifications and automated phenotyping. Nevertheless, the institutional knowledge accumulated for yeast and its ability to adapt to new experimental contexts suggest (at least to the authors) that yeast’s second century will be equally fruitful (Fig. 6). Below we highlight just a few of these nonexclusive areas for future research.

### Yeast biosensors

One underexplored aspect of yeast biotechnology lies in the field of the so-called living biosensors. Most of the tools are available to design a next-generation living dosimeter (for example) in which the sensitivity or differential sensitivity of yeast strains to an environmental stress could be used as a sensitive, unicellular canary in a coal mine. By way of example, imagine a small badge containing yeast in a semisolid medium where one strain will fluoresce green if it encounters a UV-C light source, while the control strain emits a low level of red fluorescence. An LED-based fluorimeter detects the difference and displays it on a screen or as a holographic image in the subject’s field of view. Now imagine a multiplexed badge contain dozens or hundreds of threat-specific strains. By combining this technology with a simple means to activate the badge (e.g., hydrating a lyophilized, immobilized strain set), these bio-based sensors could be particularly well suited to resource-challenged environments and autonomous, field-based applications.

Indeed, the groundwork for such autonomous applications has been laid, in large part, by the efforts of researchers who have developed increasingly sophisticated electronics to support the growth and sample collection of yeast mutants on space-based missions from Space Lab to the International Space Station to our recent Deep Space Radiation Genomics experiment (DSRG) in which the yeast deletion collections were sent to and returned from lunar orbit in 2022 [169]. This experiment represents the first long-term cosmic radiation exposure of yeast (or any biological material) in over 70 years. In parallel with these space missions, the ground-based controls will help illuminate the adverse effects induced by the complex space environment.

Finally, the idea of genetic modification of yeast (either permanently using CRISPR or transiently via controlled RNA expression) can be useful to identify genes that when modified can offer radiation resistance for long-term missions to Mars and beyond. By using the methods developed to humanize yeast to introduce diverse extremophile genes into yeast, we can directly measure the effects of genes in such extreme environments. While yeast is not a particularly extremophilic organism, with its growth limited to modest ranges of salinity, temperature, etc., its genome offers an excellent platform to systematically test exogenous transgenes for the effects of genotype on extreme environments. Indeed, much of the benchmark or control data already exists for collections of yeast mutants exposed to diverse (drugs, salt, radiation, etc.). The ability to thrive in extreme environments, to test genes for food crops, as sentinels for the effects of environmental change (temp, humidity, flooding/drought cycles, pathogens) already exists, so we have a lot of the raw material to design such experiments.

### Yeast avatars

The humanization of yeast has been employed on a gene-by-gene level to catalog a range of functional orthologs and complexes of orthologs, as well as on an allele-by-allele basis to understand the effects of both common and rare polymorphisms. The logical extension of this work will be to generate comprehensive yeast avatars for human individuals to model a range of diseases in specific genomic backgrounds. One can imagine that, by leveraging the advances in synthetic biology and genome editing, the development of a yeast avatar possessing millions of human variants in, for example, drug metabolism and cancer susceptibility genes that would accompany each of us to the pharmacy or doctor’s office.

### Robotic scientists

If a bioengineer working when Botstein and Fink published their 2011 update had suggested that, a decade later, we would be analyzing the results of fully autonomous experiments, they would be right in anticipating a cool reception. But, in fact, Steve Oliver and colleagues had already proposed a robot scientist capable of performing yeast genomic studies [170]. Recent advances in simple-to-program, inexpensive lab automation, combined with advances in natural language processing and diverse machine learning algorithms, has us on the precipice of a research community that comprises both carbon-based and silicon-based principal investigators. A case can be made that the unbiased (occasionally derided as “hypothesis-free”) nature of genomics investigations is well-suited to the “ready-fire-aim” approach used to feed machine learning applications. A useful example can be found in the discipline of in-lab evolution [171–174]. The growth characteristics of yeast make it an ideal in-lab-evolution platform, but the requirements for human intervention to decide on what traits to select for and when to impose selection are arguably better left to an algorithm that can also evolve. By simply combining optical density measurements with on-demand liquid transfers and sample collection, these assays can be maintained indefinitely. The prospect of increasing the autonomy of such a robot scientist by equipping them with LLMs to inform an autonomous analysis seems close to becoming a reality.

Regardless of the precise direction that future yeast research takes, the remarkable adaptability of this model organism is poised to remain a catalyst for groundbreaking discoveries. The unique attributes of yeast make it an invaluable tool for scientists exploring the intricacies of biological processes, ensuring that it will continue to contribute significantly to both fundamental and practical advancements in research. Its versatility not only enriches our understanding of basic biological principles but also holds the promise of impacting diverse fields, from medicine to biotechnology. In essence, the enduring legacy of yeast as a model organism lies in its capacity to inspire discoveries with far-reaching implications across the spectrum of scientific inquiry.

### Supplementary Information

**Additional file 1. **Review history.

## Glossary

ARS: Autonomously replicating sequence
ATCC: American Type Culture Collection
COSMIC: Catalogue Of Somatic Mutations In Cancer
CRIMEtoYHU: Choosing the Right Cancer-Associated Mutation for Evaluation to Yeast HUmanization
CRISPR: Clustered regularly interspaced short palindromic repeats
CRISPR-AID: Trifunctional CRISPR system comprising CRISPRa, CRISPRi, and CRISPRd
CRISPRi: CRISPR interference
CYGD: Comprehensive Yeast Genome Database
DAmP: Decreased abundance by mRNA perturbation
DDCR: DNA damage checkpoint repair
dSLAM: Diploid-based synthetic lethality analysis on microarray
HIP–HOP: Haploinsufficiency profiling–homozygous profiling
MAGESTIC: Multiplexed accurate genome editing with short, trackable, integrated cellular barcodes
MAGIC: Multifunctional genome-wide CRISPR
MAVE: Multiplexed assays of variant effect
MoBY-ORF: Molecular-barcoded yeast ORF
MSP: Multicopy suppression profiling
NGS: Next-Generation Sequencing
NHEJ: Nonhomologous end joining
PAMs: Protospacer adjacent motifs
PPIs: Protein–protein interactions
SGA: Synthetic genetic array
SGD: Saccharomyces Genome Database
SNP: Single-nucleotide polymorphisms
STRING: Search Tool for the Retrieval of Interacting Genes
TAP: Tandem affinity purification
TEV: *Tobacco etch virus*
Tn: Transposon
ts: Temperature sensitive
UMI: Unique molecular identifier
uORFs: Upstream open reading frames
Y2H: Yeast two-hybrid
YAGM: Yeast-Associated Genes Miner
YKO: Yeast knockout
YPD: Yeast Protein Database
