# Supplementary material for: From beer to breadboards: yeast as a force for biological innovation
Source: Genome Biol. 2024 Jan 4;25:10. doi: 10.1186/s13059-023-03156-9 (PMC10768129; doi:10.1186/s13059-023-03156-9)
Supplement: Supplementary file 1 — Additional file 1. Review history. [file 13059_2023_3156_MOESM1_ESM.docx]

**1^st^ round**

**Reviewer 1**

This is an informative review that attempts to cover a vast range of fields. Overall, it is generally successful. The bioinformatics and humanisation sections are particularly good. Some other sections lack detail (or in some cases perhaps have too much detail. This is always going to be a challenge in a review that covers such a wide range of fields and in general the approach of focusing in on a few specific (and generally interesting) examples works well. We have a few suggestions that we hope will help the authors improve their manuscript.


Major:

1. Figure 1: Note that here and in the main text the authors have missed two seminal papers that launched the population genomics era (at least in yeast): Liti et al. (2009) and Schacherer et al. (2009). The figure should be corrected to show this as 2009 (rather than 2011) and the main text (bottom of page 2) should be amended. Note: it is this studies that first idenfided the geographic distribution of distinct budding yeast populations (not the McCusker paper cited). In addition, the figure would benefit from citations for each of the statements.

2. I was disappointed to see so little on genome annotation. For example, ORFs were originally annotated as coding sequences of 100 codons or more. However, this included some false positives and the advent of various comparative genomics studies helped remove the false positives (and correct the boundaries of several ORFs) - in total ~10% of yeast ORF could be removed thanks to insights from comparative genomics. In addition, yeast has really led the way in functional genome annotations, in large part due to pioneering work with methods such as ChIP-seq to identify transcription factor binding sites, etc. Overall, the yeast genome is one of or possibly the best annotated genomes with many functional elements (genes, centromeres, telomeres, replication origins, protein bindings sites) identified on a truly telomere-to-telomere assembly decades before that was achieved for other eukaryotes.

3. It's not clear how Figure 3 aids understanding. It might be helped by a fuller legend. For example what do the different shapes and colours refer to? How does this figure relate to the HIP-HOP growth assay described in the text (in relation to this figure), but not mentioned in the figure or legend?

4. Figure 4a is also not particularly clear - I don't really understand what is trying to be conveyed! Perhaps a clearer legend would help.


Minor:

- abstract "seveal" should be "several"
- page 2, paragraph 2 and 3 could be merged; paragraph 2 (Starting "Owing to the...." needs a few more references
- page 2, paragraph 3: "Despite the S. cerevisiae genome is one of the best-characterized", should be "being" rather than "is".
- last paragraph on page 3, "effective" should be "effectively" - overall this sentence is cumbersome and would benefit from re-writing.
- page 6, first paragraph: "report" should be "reported"
- page 6: the structure/grammar in the following sentence needs to be corrected: "This surprising observation, i.e., that genes with altered mRNA expression in response to a given condition were not required for survival in the same condition."
- the following sentence would benefit from further explanation: "Alternatively, one can clone the drug target by complementation, identifying the clone that restores resistance, provided the mutation is recessive [79]."

**Reviewer 2**

This review presents an up-to-date overview of Brewer's yeast, Saccharomyces cerevisiae, as a model for high-throughput investigation of basic cellular processes and as a testbed for interrogation of human genes and their variants. Following a brief re-cap of the history of yeast as a model for genetics, the authors launch into a survey of the wide variety of areas in which yeast genomics has had and continues to have an impact. Their main focus is on techniques investigating gene function, both from the point of view of basic cell biology and with a view to developing models of human gene function and disease. Understandably for an article of limited length, the authors haven't given details on all of the methods/approaches they cover, but the article and its rich list of papers cited will be a valuable starting point for any researcher new to yeast or to yeast genomic approaches. That said, there are places where a little more detail would be helpful (see below). And, the manuscript would have benefited from a final, careful round of proofing, as there are many incomplete/run-on sentences that currently are tough to understand. Overall, I found this article to be very informative and I have no doubt that a beginner in the field would find it to be a terrific starting point.

Specific comments:
Please note: Throughout, I am referencing the line numbers as listed by Microsoft Word in the Word version of the document, as the line numbers shown in the pdf don't actually align with the lines of text.


1. The glossary at the beginning is very helpful, but incomplete... I don't understand why some acronyms used in the article (e.g., CTY) are listed but other acronyms (e.g., MAGIC, SWAT) are not. (And even showing "CTY" as being "CRIMEtoYHU" in the glossary is not helpful, as CRIME and YHU are not defined there.) Also, the glossary should be listed alphabetically for easier navigation.

2. p4, last paragraph: A cartoon illustrating a gene deletion along with the barcode tags (possibly as part of Figure 2) would be helpful.

3. In Figure 2, numbering the six steps to match the numbering in the figure legend would make the figure easier to follow.

4. In the description of the YKO collection (p7) it would be appropriate to also mention the caveats of using this collection, e.g., the potential for suppressors to have arisen over many rounds of serial passaging, as well as the need to perform validation (e.g., testing fresh-made knockouts of candidate genes).

5. p8, lines 15-22: The authors lay out two hypotheses for haploinsufficiency and cite one line of evidence, but stop short of stating a conclusion from the evidence. Yes, the reader can come to their own conclusions, but the authors should complete their thought and state their interpretation of the data.

6. p8, lines 10-12: Give a little bit more detail on what a colony-based study is, and what they mean by "only pathway overlapping genes".

7. p8-9, "Protein complexes identified used mass spectrometry": give a little more detail on what TAP is.

8. p9, the MoBY collection: the authors described the MoBY construct as ORFs flanked by native upstream and downstream sequences, but also mention toxicity due to overexpression. It's not clear where the overexpression comes from, if the plasmid-borne ORFs are transcribed from their native promoters. Is it just the presence of the extra copy of the ORF? A little more detail would help.

9. p10, lines 6-17: generally unclear, especially the passing reference to "genes that buffer the pathway". Since the authors mention Sir2 and tenovin, it might be helpful to step through how HIP-HOP led to the discovery of Sir2 as the target of tenovin, as an illustration of how HIP-HOP works.

10. I did not find Figure 3 to be at all useful or informative. A flowchart to illustrate the logic of HIP-HOP (along the lines of the flowchart in Figure 2) would be so much more useful.

11. p11, first paragraph: the authors should consider moving this section to follow the 2-hybrid section, as going from 2-hybrid to trigenic interaction seems a better logical flow.

12. p11, lines 25-26, "but modifying SNPs is more difficult, owing to the high degree of sequence similarity between the guide and the donor" — cryptic sentence, I couldn't figure out what the authors mean. Likewise, lines 34-38 in the same paragraph ("Taking advantage of the tri-functional CRISPR system...") gives too little detail to be useful.

13. Please consider including a diagram to illustrate barcode fusions (p11, last paragraph).

14. p13, "Humanization of Yeast": As the authors have gone to the length of explicitly defining orthologs (which most biologists would know), they should also explicitly define what a humanized yeast is (which non-yeast researchers might not know), rather than leaving it as a conclusion for the reader to make.

15. p13, lines 38-39 ("N-hydroxyurea-based PTPD against arylstibonic acid derivative NSC-13755"): cryptic, needs more detail.

16. p13, line 43: cite the reference (#134) for the work from Ed Marcotte's lab.

17. p14, last paragraph: the organization of this section is confusing, beginning with an example and then stating what the problem is. The second sentence ("For numerous complex interactions...") is particularly confusing. Perhaps lead off the paragraph with these thoughts, then give the example? Maybe something like: "Numerous complex interactions with various organelles and signaling pathways that are present in human cells are absent in yeast. In these instances, model, humanized strains can be used to examine the pathway in a 'clean' background. For example, Boonekamp et al. recently reported the first successful humanization of skeletal muscle glycolysis in yeast, opening up the possibility of exploration of human glycolysis in yeast [141]. When paired with evolutionary strategies, single gene and complete pathway transplanting demonstrated the extraordinary conservation of glycolytic and moonlighting functions and indicated context-dependent responses. For instance, the study showed that human hexokinases 1 and 2, but not 4, needed alterations in their catalytic or allosteric regions in order to operate in yeast, while hexokinase 3 was unable to complement its yeast ortholog. Human glycolytic enzyme turnover rates were preserved in yeast and human cell cultures when compared to human tissues. The construction of metazoan models that are tailored to certain species, tissues, and diseases is made possible by this example of the transplanting of a complete critical pathway [141]. However, the characterization of the physiological and cellular impact of the transplantation is one of these techniques' general limitations."

18. p15, lines 46-48: CRISPR has already been mentioned much earlier in the article, that would be the appropriate place for this sentence regarding the history of CRISPR technology. The organization of this paragraph could be improved: currently, it talks about pathway reconstitution, then CRISPR, then goes back to pathways. The connections are unclear.

19. Reference #155 (p18, line 1) is an incorrect citation. The one listed ("Make way for robot scientists") is a rebuttal letter in response to a letter critiquing the original article. The correct citation is DOI: 10.1126/science.1165620. (I did not bother to check all citations; the authors should go through and verify that they are all correct.)

Typos/proofing errors:

1. Abstract: ancestors, not ancestor's.

2. p1, line 39: Herschel, not Hershel.

3. p2, line 22: something wrong with this sentence.

4. p2, line 35: should be hundreds (plural).

5. p3, lines 14-15 ("When the yeast sequencing project completed, assigning functions effective means to test to newly discovered gene sequences became a priority")—the sentence doesn't make sense.

6. p4, line 1: "now the genome", not "how the genome"?

7. p4, line 31 ("cassette was transformed into a diploid strain")—jargon; the cassette isn't transformed, it's the strain that is transformed. Better to say "cassette was introduced into a diploid strain".

8. p4, line 39: remove parentheses around UMIs.

9. p6, line 1: progress report, not report progress.

10. p6, lines 15-16 ("This surprising observation, i.e., that genes with altered mRNA expression in response to a given condition were not required for survival in the same condition.") Incomplete sentence/thought.

11. p6, line 34: italicise Arabidopsis thaliana. Likewise with Drosophila (p7 line 26) and gene names such as SEC14 (p11 line 30).

12. p7, line 2 ("Using these collections allowed them"): unclear who "them" refers to.

13. p8 line 42: still, not till.

14. p11, end of 2nd paragraph ("The gold standard online resource for data and annotation SGD webpage (yeastgenome.org).") Incomplete sentence, perhaps a translocation: a fuller version of this sentence appears in the first paragraph of Section 5, Bioinformatics.

15. p11, line 29: unclear what "to aid study variants" means... to aid the study of variants?

16. p14, line 23: Niemann-Pick, not -Pic.

17. p15, line 20: remove period after "desired behavior".

18. End of p.15 ("In 2013, Church's team reported a CRISPR-based approach for site-specific mutagenesis and allelic replacement in yeast, where constitutive expression of Cas 9 and a gRNA plasmid and donor DNA in very high recombination frequency [149].")—incomplete sentence. Also, Cas9, not Cas 9.

19. p17, line 35: "ability" should not be capitalized.

20. p18, line 4: missing closing quotes around hypothesis-free.

**Authors’ response**

Reviewer 1

Major:

1.Figure 1: Note that here and in the main text the authors have missed two seminal papers that launched the population genomics era (at least in yeast): Liti et al. (2009) and Schacherer et al. (2009). The figure should be corrected to show this as 2009 (rather than 2011) and the main text (bottom of page 2) should be amended. Note: it is this studies that first idenfided the geographic distribution of distinct budding yeast populations (not the McCusker paper cited). In addition, the figure would benefit from citations for each of the statements.

Thank you and we apologies for our oversight. This section has been completely rewritten to reflect the significance of these studies in yeast population genomics.

2. I was disappointed to see so little on genome annotation. For example, ORFs were originally annotated as coding sequences of 100 codons or more. However, this included some false positives and the advent of various comparative genomics studies helped remove the false positives (and correct the boundaries of several ORFs) - in total ~10% of yeast ORF could be removed thanks to insights from comparative genomics. In addition, yeast has really led the way in functional genome annotations, in large part due to pioneering work with methods such as ChIP-seq to identify transcription factor binding sites, etc. Overall, the yeast genome is one of or possibly the best annotated genomes with many functional elements (genes, centromeres, telomeres, replication origins, protein bindings sites) identified on a truly telomere-to- telomere assembly decades before that was achieved for other eukaryotes.

This is an important point and we have added a section on pages 2 and 3 to underscore the importance of yeast in gene/genome annotation.

3. It's not clear how Figure 3 aids understanding. It might be helped by a fuller legend. For example what do the different shapes and colours refer to? How does this figure relate to the HIP-HOP growth assay described in the text (in relation to this figure), but not mentioned in the figure or legend?

4. Figure 4a is also not particularly clear - I don't really understand what is trying to be conveyed! Perhaps a clearer legend would help.

Figures 2, 3 and 4 have been updated to reflect your suggestions.

Minor:

- abstract "seveal" should be "several"

Corrected

- page 2, paragraph 2 and 3 could be merged; paragraph 2 (Starting "Owing to the...." needs a few more references

Corrected text and provided additional references.

- page 2, paragraph 3: "Despite the S. cerevisiae genome is one of the best- characterized", should be "being" rather than "is".

Corrected

- last paragraph on page 3, "effective" should be "effectively" - overall this sentence is cumbersome and would benefit from re-writing.

The paragraph has been rewritten

- page 6, first paragraph: "report" should be "reported"

Corrected

- page 6: the structure/grammar in the following sentence needs to be corrected: "This surprising observation, i.e., that genes with altered mRNA expression in response to a given condition were not required for survival in the same condition."

Thank you, we redrafted this sentence as follows’… The 2002 study also established that there is little correlation between those genes that are required for fitness in a particular condition (represented by their corresponding deletion strain) and the transcriptional change in that same gene’s transcript in response to the same condition [34].

- the following sentence would benefit from further explanation: "Alternatively, one can clone the drug target by complementation, identifying the clone that restores resistance, provided the mutation is recessive [79]."

Clarified and edited to read, Traditional chemogenomic approaches to determine the mechanism of action (MoA) of drugs include isolation of drug-resistant mutants folloed my genetic mapping. While this mutational approach can identify the drug target, it is difficult to scale. Alternatively, one can clone a drug target by complementation [98]. In one early cloning-by-complementation study, a strain mutated for the gene encoding a drug target (HMG-CoA reductase) was transformed with a genomic DNA clone bank, to identify drug-resistant colonies able to grow on solid media containing Lovastatin [99]. This method inspired Multicopy Suppression Profiling (MSP), where a library of clones is introduced, in parallel, into a pool of mutants and once resistant strains are identified, the complementing plasmid-borne gene is sequenced to reveal candidate drug targets.

Reviewer 2

And, the manuscript would have benefited from a final, careful round of proofing, as there are many incomplete/run-on sentences that currently are tough to understand. Apologies for these grammatical and typographical errors. They have been corrected and the entire manuscript has been extensively copyedited and shortened by 1000 words.

1. The glossary at the beginning is very helpful, but incomplete... I don't understand why some acronyms used in the article (e.g., CTY) are listed but other acronyms (e.g., MAGIC, SWAT) are not. (And even showing "CTY" as being "CRIMEtoYHU" in the glossary is not helpful, as CRIME and YHU are not defined there.) Also, the glossary should be listed alphabetically for easier navigation.

Thank you for this suggestion. We have expanded the glossary and ordered it alphabetically.

2. p4, last paragraph: A cartoon illustrating a gene deletion along with the barcode tags (possibly as part of Figure 2) would be helpful.

Thank you, we have added a panel to Figure 2 to illustrate the deletion strategy.

3. In Figure 2, numbering the six steps to match the numbering in the figure legend would make the figure easier to follow.

Agreed and corrected

4. In the description of the YKO collection (p7) it would be appropriate to also mention the caveats of using this collection, e.g., the potential for suppressors to have arisen over many rounds of serial passaging, as well as the need to perform validation (e.g., testing fresh-made knockouts of candidate genes).

We have added the following cautionary paragraph, “Despite its merits, the YKO has several key limitations that limit its utility. These include the fact that the YKO represents a single genetics background, which contains several well-characterized polymorphism that compromise sporulation, mitochondrial function and other less obvious phenotypes [85]. While there are other yeast deletion mutant collections, most of these are hybrids in which the deletion cassettes were derived from the original YKO (for example [84] and [86]). Improper maintenance of large strain collections (wrong colony on plate or polyclonal colonies) can lead to the wrong mutant being tested. Another confounding factor is that individual mutant strains may have collected mutations (e.g. second-site suppressors, aneuploidy, and diploidization). Accordingly, it is recommended that results from YKO experiments (either individually or in pools) should be independently constructed and validated. The compact nature of the yeast genome often complicates the study of individual genes. Specifically, deletion of the ORF of one gene can disrupt the promoter, terminator or coding sequences of nearby genes on the opposite strand of genomic DNA.”

5. p8, lines 15-22: The authors lay out two hypotheses for haploinsufficiency and cite one line of evidence, but stop short of stating a conclusion from the evidence. Yes, the reader can come to their own conclusions, but the authors should complete their thought and state their interpretation of the data.

We have revised this description as follows, “Taken together, this work suggests that most cases of haploinsufficiency in yeast is caused by insufficient amounts of protein, with some exceptions like cytoskeletal genes (ACT1, TUB1, and SPC97) that maintain haploinsufficiency in both YPD and minimal media; for those genes, the balance hypothesis best explains their haploinsufficiency [73].”

6. p8, lines 10-12: Give a little bit more detail on what a colony-based study is, and what they mean by "only pathway overlapping genes".

Revised to include, “As opposed to liquid growth assays that typically measure fitness by light scattering, colony-based yeast studies measure fitness based on colony size.”

7. p8-9, "Protein complexes identified used mass spectrometry": give a little more detail on what TAP is.

Corrected as suggested

8. p9, the MoBY collection: the authors described the MoBY construct as ORFs flanked by native upstream and downstream sequences, but also mention toxicity due to overexpression. It's not clear where the overexpression comes from, if the plasmid- borne ORFs are transcribed from their native promoters. Is it just the presence of the extra copy of the ORF? A little more detail would help. Toxicity mainly from Gal, limited tox from increasing gene copy number

Apologies for conflating the issues of copy number and expression level. We simplified the description as follows, “This collection is barcoded, and because each CEN-based plasmid carries a single ORF flanked by its native upstream and downstream genomic sequences, the copy number is low and predictable, minimizing overexpression toxicity [102]. Indeed, while over-expression can disrupt cellular homeostasis, several groups have exploited this phenotype to find inhibitors that alleviate the fitness defect caused by overexpression of toxic proteins [103–105].”

9. p10, lines 6-17: generally unclear, especially the passing reference to "genes that buffer the pathway". Since the authors mention Sir2 and tenovin, it might be helpful to step through how HIP-HOP led to the discovery of Sir2 as the target of tenovin, as an illustration of how HIP-HOP works.

Apologies. We clarified this section to read, “An illustrative example includes Sir2, a histone deacetylase, as the target of tenovin, a small-molecule p53 activator [112] This study screened the heterozygous diploid deletion with a derivative of tenovir to show that Sir2 manifested tenovir-induced haploinsufficiency. While none of the Silent Information Regulator genes where not represented in the YKO (because they are unable to mate) the deletion of an adjacent dubious ORF (YDL041W) removed the first 300 nucleotides of SIR2, abolishing its function and establishing Sir2 as a potential tenovir target.”

10. I did not find Figure 3 to be at all useful or informative. A flowchart to illustrate the logic of HIP-HOP (along the lines of the flowchart in Figure 2) would be so much more useful.

Both Figures 2 and 3 have been substantially revised.

11. p11, first paragraph: the authors should consider moving this section to follow the 2-hybrid section, as going from 2-hybrid to trigenic interaction seems a better logical flow.

This section has been extensively copyedited.

12. p11, lines 25-26, "but modifying SNPs is more difficult, owing to the high degree of sequence similarity between the guide and the donor" — cryptic sentence, I couldn't figure out what the authors mean. Likewise, lines 34-38 in the same paragraph ("Taking advantage of the tri-functional CRISPR system...") gives too little detail to be useful.

This section has been extensively copyedited.

13. Please consider including a diagram to illustrate barcode fusions (p11, last paragraph).

We have included added a new figure 4.

14. p13, "Humanization of Yeast": As the authors have gone to the length of explicitly defining orthologs (which most biologists would know), they should also explicitly define what a humanized yeast is (which non-yeast researchers might not know), rather than leaving it as a conclusion for the reader to make.

This section has been extensively copyedited.

15. p13, lines 38-39 ("N-hydroxyurea-based PTPD against arylstibonic acid derivative NSC-13755"): cryptic, needs more detail.

Revised to read, “The Hieter laboratory used this system to test two known human FEN1 inhibitors; the N-hydroxyurea-based compound PTPD and a derivative of NSC- 13755. While both compounds inhibited growth of a humanized hFEN1 yeast strain in the presence of MMS, PTPD was a potent, specific inhibitor of hFEN1 while NSC- 13755 was associated with general toxicity [151].”

16. p13, line 43: cite the reference (#134) for the work from Ed Marcotte's lab.

Corrected

17. p14, last paragraph: the organization of this section is confusing, beginning with an example and then stating what the problem is. The second sentence ("For numerous complex interactions...") is particularly confusing. Perhaps lead off the paragraph with these thoughts, then give the example? Maybe something like: "Numerous complex interactions with various organelles and signaling pathways that are present in human cells are absent in yeast. In these instances, model, humanized strains can be used to examine the pathway in a 'clean' background. For example, Boonekamp et al. recently reported the first successful humanization of skeletal muscle glycolysis in yeast, opening up the possibility of exploration of human glycolysis in yeast [141]. When paired with evolutionary strategies, single gene and complete pathway transplanting demonstrated the extraordinary conservation of glycolytic and moonlighting functions and indicated context-dependent responses. For instance, the study showed that human hexokinases 1 and 2, but not 4, needed alterations in their catalytic or allosteric regions in order to operate in yeast, while hexokinase 3 was unable to complement its yeast ortholog. Human glycolytic enzyme turnover rates were preserved in yeast and human cell cultures when compared to human tissues. The construction of metazoan models that are tailored to certain species, tissues, and diseases is made possible by this example of the transplanting of a complete critical pathway [141]. However, the characterization of the physiological and cellular impact of the transplantation is one of these techniques' general limitations."

Corrected as suggested.

18. p15, lines 46-48: CRISPR has already been mentioned much earlier in the article, that would be the appropriate place for this sentence regarding the history of CRISPR technology. The organization of this paragraph could be improved: currently, it talks about pathway reconstitution, then CRISPR, then goes back to pathways. The connections are unclear.

This section has been extensively copyedited.

19. Reference #155 (p18, line 1) is an incorrect citation. The one listed ("Make way for robot scientists") is a rebuttal letter in response to a letter critiquing the original article. The correct citation is DOI: 10.1126/science.1165620. (I did not bother to check all citations; the authors should go through and verify that they are all correct.)

This entire manuscript has been extensively copyedited and each reference has been double-checked.

Typos/proofing errors:

Each of these have been corrected thank you.

1. Abstract: ancestors, not ancestor's.

2. p1, line 39: Herschel, not Hershel.

3. p2, line 22: something wrong with this sentence.

4. p2, line 35: should be hundreds (plural).

5. p3, lines 14-15 ("When the yeast sequencing project completed, assigning functions effective means to test to newly discovered gene sequences became a priority")—the sentence doesn't make sense.

6. p4, line 1: "now the genome", not "how the genome"?

7. p4, line 31 ("cassette was transformed into a diploid strain")—jargon; the cassette isn't transformed, it's the strain that is transformed. Better to say "cassette was introduced into a diploid strain".

8. p4, line 39: remove parentheses around UMIs.

9. p6, line 1: progress report, not report progress.

10. p6, lines 15-16 ("This surprising observation, i.e., that genes with altered mRNA expression in response to a given condition were not required for survival in the same condition.") Incomplete sentence/thought.

11. p6, line 34: italicise Arabidopsis thaliana. Likewise with Drosophila (p7 line 26) and gene names such as SEC14 (p11 line 30).

12. p7, line 2 ("Using these collections allowed them"): unclear who "them" refers to.

13. p8 line 42: still, not till.

14. p11, end of 2nd paragraph ("The gold standard online resource for data and

annotation SGD webpage (yeastgenome.org).") Incomplete sentence, perhaps a translocation: a fuller version of this sentence appears in the first paragraph of Section 5, Bioinformatics.

15. p11, line 29: unclear what "to aid study variants" means... to aid the study of variants?

16. p14, line 23: Niemann-Pick, not -Pic.

17. p15, line 20: remove period after "desired behavior".

18. End of p.15 ("In 2013, Church's team reported a CRISPR-based approach for site- specific mutagenesis and allelic replacement in yeast, where constitutive expression of Cas 9 and a gRNA plasmid and donor DNA in very high recombination frequency [149].")—incomplete sentence. Also, Cas9, not Cas 9.

19. p17, line 35: "ability" should not be capitalized.

20. p18, line 4: missing closing quotes around hypothesis-free.

**2^nd^ round**

**Reviewer 1**

The authors have done a good job of addressing referee comments and the resulting manuscript is an improvement. I have a number of more minor comments listed below:


1. Page 2 - define ORF at first use.

2. Check italics throughout, e.g. Saccharomyces cerevisiae in table 1 legend, SIR2 on page 11.

3. I remain uncomfortable about the lack of citations associated with figure 1.

4. Page 5: "with the KanMX deletion ‘cassette’" - refer to figure 2a here.

5. Figure 2b: the final panel (6) alters the color order compared to all the other panels.

6. The paragraph on limitations of the YKO is under the section heading 'Mitochondrial respiration screens' - I think it might fit better at the end of the previous section 'Genome-wide phenotypic screens'.

7. Page 10: "followed my genetic mapping" *my* should be *by*.

8. Check for consistency of "SGD" vs "the SGD".

9. Page 15: “Orthologs are genes originating from a single ancestral gene in the last common ancestor of the compared genomes” [146]. If a whole sentence is quoted from a paper, perhaps credit the authors names in the text. E.g. Koonin defines orthologs as "genes originating from a single ancestral gene in the last common ancestor of the compared genomes” [146].

10. Page 15: should "Humanized" be capitalised?

11. Page 15: which genes names should be italicised in "FEN1 (human ortholog of in yeast)" - certainly the yeast gene name, RAD27, should be italicised.

12. Page 17: "impact of the transplantation is one of these techniques' general limitations."" - some strange use of quotations (single and double) in the text.

13. I can't find where figure 6 is referred to in the main text.

14. Page 20: "...us to the Pharmacy or Doctor’s office." - check for appropriate use of capitalisation.

15. I wasn't convinced that the final paragraph using ChatGPT added to the manuscript - perhaps a few extra sentences to set the context and speculate as to how these technologies may expand research directions?

16. Page 20: define LLM.

**Reviewer 2**

The revised manuscript is greatly improved and I think it addresses all my previous major concerns. The issues I have listed below are all very minor and are almost all just typographical errors. (Line numbering in the left margins does not actually align with the lines of text, so the line numbers I list below are approximate.)

Minor comments
1. p2, Table 1 legend: the last sentence of the legend is incomplete ("...a recent study using long-read, single molecule sequence provided a telomere-to-telomere [11].")

2. p3, line 27 (very minor!): chromatin immunoprecipitation is usually abbreviated as ChIP, not CHIP.

3. p5, line 15: should be start-to-stop deletions, not start-to-start

lines 23-24: missing a closing apostrophe for 'molecular barcodes'

4. p9: lines 29-31 (discussion of limitations of YKO collections) is redundant with lines 32-34.

line 33: should be genetic background, not genetics background

5. p10 line 20/21: delete the comma ("Newer protein-protein interaction technologies,...")

line 45: should be "followed by genetic mapping" and not "followed my..."

6. p11 line 22: "HIP uses essential heterozygous deletion strains in competitive fitness assays combined with quantitative analysis of the molecular barcodes identifies relative strain abundance" ... something wrong with this sentence -- should be "to identify relative strain abundance", I think.

line 37/38: "While none of the Silent Information Regulator genes where not represented..." -- should be, "none of the genes... were represented"

7. p14 line 14/15: remove the comma after Bioinformatics ("Bioinformatics, played a vital role...")

line 28: Language Models, not Lange Models

lines 43-48: redundant sentences about GeneMANIA

8. p15 line 46/47: missing a full-stop at the end of the sentence.

9. p17 line 25/26: remove the double-quote at the end of the paragraph

lines 47-48: very long run-on sentence with multiple semi-colons. Consider breaking them up into separate sentences.

10. p18 line 13: the term "developing countries" can be controversial (the World Bank no longer uses the term)... I don't have an alternative except perhaps "low gross national income". It's just something to be aware of.

11. p19 line 36/37: should be medium, not media.

**Authors’ response**

Page 2 - define ORF at first use.

corrected

Check italics throughout, e.g. Saccharomyces cerevisiae in table 1 legend, SIR2 on page 11.

corrected throughout

3. I remain uncomfortable about the lack of citations associated with figure 1.

corrected

4. Page 5: "with the KanMX deletion ‘cassette’" - refer to figure 2a here.

corrected

5. Figure 2b: the final panel (6) alters the color order compared to all the other panels

Thank you for pointing this out. The change was made to accommodate the human- yeast comparison.

6. The paragraph on limitations of the YKO is under the section heading 'Mitochondrial respiration screens' - I think it might fit better at the end of the previous section 'Genome-wide phenotypic screens'.

Thank you for this suggestion, and we did consider this change. We opted to keep the “limitations” in the earlier description of the mitochondrial screens, so that the reader would have them in mind when we introduced the more complex phenotypic screens.

7. Page 10: "followed my genetic mapping" *my* should be *by*.

corrected

8. Check for consistency of "SGD" vs "the SGD". (

corrected throughout

9. Page 15: “Orthologs are genes originating from a single ancestral gene in the last common ancestor of the compared genomes” [146]. If a whole sentence is quoted from a paper, perhaps credit the authors names in the text. E.g. Koonin defines orthologs as "genes originating from a single ancestral gene in the last common ancestor of the compared genomes” [146].

Excellent point, corrected

10. Page 15: should "Humanized" be capitalised?

Based on usage in the primary literature, we opted to keep humanized lower case.

11. Page 15: which genes names should be italicised in "FEN1 (human ortholog of in yeast)" - certainly the yeast gene name, RAD27, should be italicised.

Thank you, corrected as suggested

12. Page 17: "impact of the transplantation is one of these techniques' general limitations."" - some strange use of quotations (single and double) in the text.

corrected

13. I can't find where figure 6 is referred to in the main text. (I mentioned that in “Three areas to watch“)

corrected

14. Page 20: "...us to the Pharmacy or Doctor’s office." - check for appropriate use of capitalisation.

Thank you, this appears to be regionally variable.

15. I wasn't convinced that the final paragraph using ChatGPT added to the manuscript

- perhaps a few extra sentences to set the context and speculate as to how these technologies may expand research directions?

Thank you for the suggestion, we agree that section does not add significantly and have deleted it.

16. Page 20: define LLM.

Corrected

Reviewer 2

Minor comments

1.p2, Table 1 legend: the last sentence of the legend is incomplete ("...a recent study using long-read, single molecule sequence provided a telomere-to-telomere [11].")

corrected

2.p3, line 27 (very minor!): chromatin immunoprecipitation is usually abbreviated as ChIP, not CHIP.

corrected

3. p5, line 15: should be start-to-stop deletions, not start-to-start

corrected

lines 23-24: missing a closing apostrophe for 'molecular barcodes'

corrected

4. p9: lines 29-31 (discussion of limitations of YKO collections) is redundant with lines 32-34.

rewritten

line 33: should be genetic background, not genetics background

corrected

5. p10 line 20/21: delete the comma ("Newer protein-protein interaction technologies,...")

corrected

line 45: should be "followed by genetic mapping" and not "followed my..."

corrected

6. p11 line 22: "HIP uses essential heterozygous deletion strains in competitive fitness assays combined with quantitative analysis of the molecular barcodes identifies relative strain abundance" ... something wrong with this sentence -- should be "to identify relative strain abundance", I think.

corrected

line 37/38: "While none of the Silent Information Regulator genes where not represented..." -- should be, "none of the genes... were represented"

corrected

7. p14 line 14/15: remove the comma after Bioinformatics ("Bioinformatics, played a vital role...")

corrected

line 28: Language Models, not Lange Models

corrected

lines 43-48: redundant sentences about GeneMANIA

corrected

8. p15 line 46/47: missing a full-stop at the end of the sentence.

corrected

9. p17 line 25/26: remove the double-quote at the end of the paragraph

Corrected

lines 47-48: very long run-on sentence with multiple semi-colons. Consider breaking them up into separate sentences.

corrected

10. p18 line 13: the term "developing countries" can be controversial (the World Bank no longer uses the term)... I don't have an alternative except perhaps "low gross national income". It's just something to be aware of.

corrected to read- lower income

11. p19 line 36/37: should be medium, not media.

corrected
